# Harnessing rhizosphere microbes: the synergistic roles of PGPR and AMF in sustainable tomato production under stress

**DOI:** 10.3389/fmicb.2026.1746930

**Published:** 2026-03-19

**Authors:** Yumei Shi, Honglong Chu, Ruoxin He, Wenjie Ma, Qilong Liang, Zhumei Li, Yong Gao, Changxin Luo

**Affiliations:** College of Biology and Food Engineering, Qujing Normal University, Qujing, Yunnan, China

**Keywords:** AMF, PGPR, rhizospheric microbiome, stress tolerance, sustainable agriculture, tomato

## Abstract

Tomato (*Lycopersicon esculentum* L.) is among the most economically important vegetable crops worldwide, yet its production is severely constrained by multiple biotic and abiotic stresses, including pathogens, pests, drought, salinity, and heavy metal toxicity. Amid intensifying climate change and increasing demands for sustainable agriculture, plant growth-promoting rhizobacteria (PGPR) and arbuscular mycorrhizal fungi (AMF) have emerged as key beneficial rhizospheric microorganisms with significant potential for enhancing plant stress tolerance and promoting growth. PGPR directly promote the growth of tomato plants through biological nitrogen fixation, solubilization of phosphate and potassium, siderophore-mediated iron uptake, and the production of phytohormones. Indirectly, PGPR suppress pathogens, activate induced systemic resistance (ISR), reinforce cell walls, enhance the activities of antioxidant enzymes, and regulate the accumulation of osmolytes. AMF form symbiotic associations with the roots of tomato plants, enhancing nutrient and water absorption via extraradical mycelial networks, improving phosphorus and nitrogen uptake, modulating abscisic acid (ABA), jasmonic acid (JA), and strigolactone signaling pathways, activating mycorrhiza-induced resistance (MIR), and enhancing photosynthetic efficiency and water-use efficiency under stress. The co-inoculation of PGPR and AMF yields synergistic effects by facilitating mutual colonization, optimizing nutrient bioavailability, coordinately strengthening antioxidant and osmotic regulation systems, and reinforcing systemic defense responses, thereby conferring more robust and efficient stress tolerance than single inoculations. Despite significant advances, key challenges persist in elucidating tripartite molecular crosstalk, maintaining stability during field applications, and developing tailored microbial consortia. This review synthesizes the individual and synergistic mechanisms through which PGPR and AMF enhance the resilience of tomato plants to biotic and abiotic stresses, offering valuable insights for engineering microbial communities to enhance stress resistance in crops.

## 1 Introduction

Tomato (*Lycopersicon esculentum* L.) is an economically important crop cultivated worldwide. According to the Food and Agriculture Organization (FAO), the global production of fresh tomatoes reached approximately 192 million tonnes in 2023. The consumption of tomatoes continues to rise worldwide, driven by demand from both the fresh market and the processed food industry (Liu et al., 2022; Li Q. et al., 2023). The widespread consumption of this fruit stems from its high nutritional value, being a rich source of bioactive compounds, including carotenoids such as lycopene and β-carotene, phenolic substances such as flavonoids, and essential vitamins such as ascorbic acid (vitamin C), α-tocopherol (vitamin E), and vitamin A (Kumar et al., 2021; Naik et al., 2023). Additionally, tomatoes contain other functional constituents, including glycoalkaloids such as tomatine, as well as diverse phytosterols, including β-sitosterol, campesterol, and stigmasterol (Kumar et al., 2021). Among these, lycopene is the primary pigment responsible for the characteristic red color of ripe tomatoes and is known for its potent antioxidant properties. Tomatoes serve as the principal commercial source of lycopene, supplying approximately 80% of the global industrial demand (Kumar et al., 2021; Tufail et al., 2024). Nevertheless, tomato cultivation is increasingly challenged by a range of abiotic and biotic stressors. The abiotic stressors include drought, salinity, temperature extremes, and nutrient deficiencies, whereas biotic stresses arise from infestations by pests and pathogens, including insects, fungi, bacteria, nematodes, and viruses (Soltabayeva et al., 2022; Li W. et al., 2023; Shi et al., 2025). Collectively, these stresses impair plant growth, development, and yield. Climate change has further exacerbated the frequency and intensity of abiotic stresses—particularly drought, heat, salinity, and flooding—thereby threatening future crop productivity (Zhang et al., 2014; Rai et al., 2022; Shawky et al., 2024). Drought stress, in particular, triggers complex molecular and cellular responses that affect nearly all physiological processes (Zhang et al., 2014). Water scarcity affects the majority of plant functions through direct or indirect mechanisms. Despite species-specific variations, plants generally perceive environmental stress signals and transduce them internally to activate appropriate defense mechanisms (Golldack et al., 2014). To mitigate drought effects, plants adjust root architecture, enhance osmotic regulation, optimize water-use efficiency (WUE), enhance the activities of antioxidant enzymes, and reduce stomatal conductance (Gs) to conserve water (Sun et al., 2020). These stress responses are largely mediated by phytohormones and signaling molecules, with abscisic acid (ABA) being the most extensively studied phytohormone in the context of abiotic stress, particularly drought. The levels of ABA rise rapidly under stress and play a central role in regulating stomatal closure, plant growth suppression, and the activation of defense pathways (Peleg and Blumwald, 2011; Osakabe et al., 2014; Muhammad Aslam et al., 2022). Beyond intrinsic defense mechanisms, plants establish symbiotic associations with rhizospheric microorganisms to alleviate stress. These include naturally occurring or deliberately introduced microbes, such as plant growth-promoting rhizobacteria (PGPR) and arbuscular mycorrhizal fungi (AMF), both of which have been shown to significantly enhance plant resilience under adverse conditions (Ton et al., 2009; Ruiz-Lozano et al., 2016; Han Y. et al., 2022).

PGPR and AMF, including strains applied to mitigate salinity stress, play vital roles in enhancing plant survival under adverse conditions (Li W. et al., 2023; Mazumder et al., 2025). Certain PGPR function as biological control agents by inhibiting spoilage organisms and offer a safer alternative to synthetic agrochemicals that may pose risks to human health, livestock, and beneficial soil microbes (Adedayo et al., 2022). The application of AMF provides an effective and environmentally sustainable strategy for mitigating the negative effects of abiotic stresses, including salinity, drought, temperature extremes, and heavy metal toxicity (Chandrasekaran et al., 2021). These microorganisms support plant growth, activate defense responses, enhance stress tolerance, and contribute to successful fruit development. This review comprehensively examines the roles of PGPR and AMF in promoting the health of tomato plants, with particular emphasis on the regulatory mechanisms underlying resistance to biotic and abiotic stressors.

## 2 Microbial influences on plant stress responses

Over hundreds of millions of years of co-evolution, plants have developed intricate associations with a diverse array of microorganisms, including bacteria, fungi, archaea, protists, and viruses (Gong and Xin, 2021). Bacteria and fungi constitute the majority of microbial biomass within these communities. Such symbiotic interactions have been fundamental in shaping plant adaptability to both biotic and abiotic stresses (Gong and Xin, 2021; Bai et al., 2022; Xiong and Lu, 2022). The assembly and stability of these microbial communities are governed by host plant selection, microbial dispersal from surrounding environments, and interspecies interactions (Trivedi et al., 2020; Zeng et al., 2025). Under stress conditions, plants alter their metabolism and root exudation patterns, thereby reshaping the composition and function of associated microbial communities to enhance stress tolerance (Santos-Medellín et al., 2017; Timm et al., 2018; Ge et al., 2023). These plant–microbe interactions contribute to the establishment of specialized ecological niches that facilitate microbial colonization and promote host growth and defense capacity (Stringlis et al., 2018). Plants actively recruit beneficial microbes under biotic stress to suppress the growth of pathogens (Ge et al., 2023). Plant-associated microbiomes form highly interactive ecological networks that become more robust under environmental stress conditions (Soliveres et al., 2014; Toju et al., 2018). Beneficial microorganisms compete with pathogens for critical resources, including siderophores and essential nutrients, thereby suppressing pathogen proliferation and reducing virulence (Raaijmakers and Mazzola, 2012). In disease-suppressive soils, the roots of sugar beet are enriched with microbial families such as *Chitinophagaceae* and *Flavobacteriaceae*, which express defense-related enzymes—including chitinase, nonribosomal peptide synthetase (NRPS), and polyketide synthase (PKS)—in response to pathogen attacks, thereby conferring resistance against *Rhizoctonia solani* (Mendes et al., 2011; Chapelle et al., 2016; Carrión et al., 2018). Similarly, resident soil bacteria suppress the growth of *Ralstonia solanacearum* through competition for essential resources. In the rhizospheres of healthy tomato plants, microbial communities are enriched in beneficial taxa, including *Actinobacteria* and *Firmicutes*, which comprise numerous biocontrol agents (Wei et al., 2015; Su et al., 2020). It has been reported that species with the genera *Bacillus* and *Pseudomonas* produce antibiotics with antibacterial, insecticidal, and antiviral activities. By occupying ecological niches and restricting pathogen access to nutrients, these beneficial microorganisms contribute to plant health and are widely used in biocontrol strategies (Zelezniak et al., 2015; Koprivova et al., 2019). In *Arabidopsis thaliana*, infection by *Hyaloperonospora arabidopsidis* or *Botrytis cinerea* induces the recruitment of specific bacterial consortia that enhance plant defense responses (Berendsen et al., 2018). In summary, beneficial microorganisms play a pivotal role in mitigating biotic stress through direct competition with pathogens and the production of antimicrobial compounds, thereby enhancing plant fitness and reducing disease incidence.

Plants can also enhance their growth under abiotic stress by recruiting beneficial microorganisms (Ge et al., 2023), which contribute to drought tolerance by enhancing the activities of antioxidant enzymes, improving WUE, modulating the production of phytohormones such as ABA, and facilitating nutrient uptake. In response to drought, microbial communities restructure their composition to support plant recovery, thereby enhancing adaptability and productivity under conditions of water limitation (de Vries et al., 2020). In rice, drought conditions significantly alter both bacterial and fungal communities in the rhizosphere and endosphere, thereby favoring the enrichment of taxa such as *Actinobacteria* and *Chloroflexi*, while reducing the abundance of *Acidobacteria* and *Deltaproteobacteria*. These shifts suggest that drought conditions selectively modulate the rhizospheric microbiome to enhance plant survival (Santos-Medellín et al., 2017). Under aluminum stress, aluminum-tolerant soybean genotypes recruit diverse microbial taxa, including *Tumebacillus*, *Granulicella*, and *Burkholderia*, which enhance plant resistance by increasing the activities of soil enzymes and reshaping bacterial community structure (Lian et al., 2019). Similarly, methylotrophic bacteria, including *Methylobacterium oryzae* and *Burkholderia* spp., can reduce the accumulation of heavy metals such as nickel and cadmium in tomato plants by restricting metal uptake and translocation, while concurrently suppressing stress-induced ethylene emissions (Madhaiyan et al., 2007).

**Figure 1:**
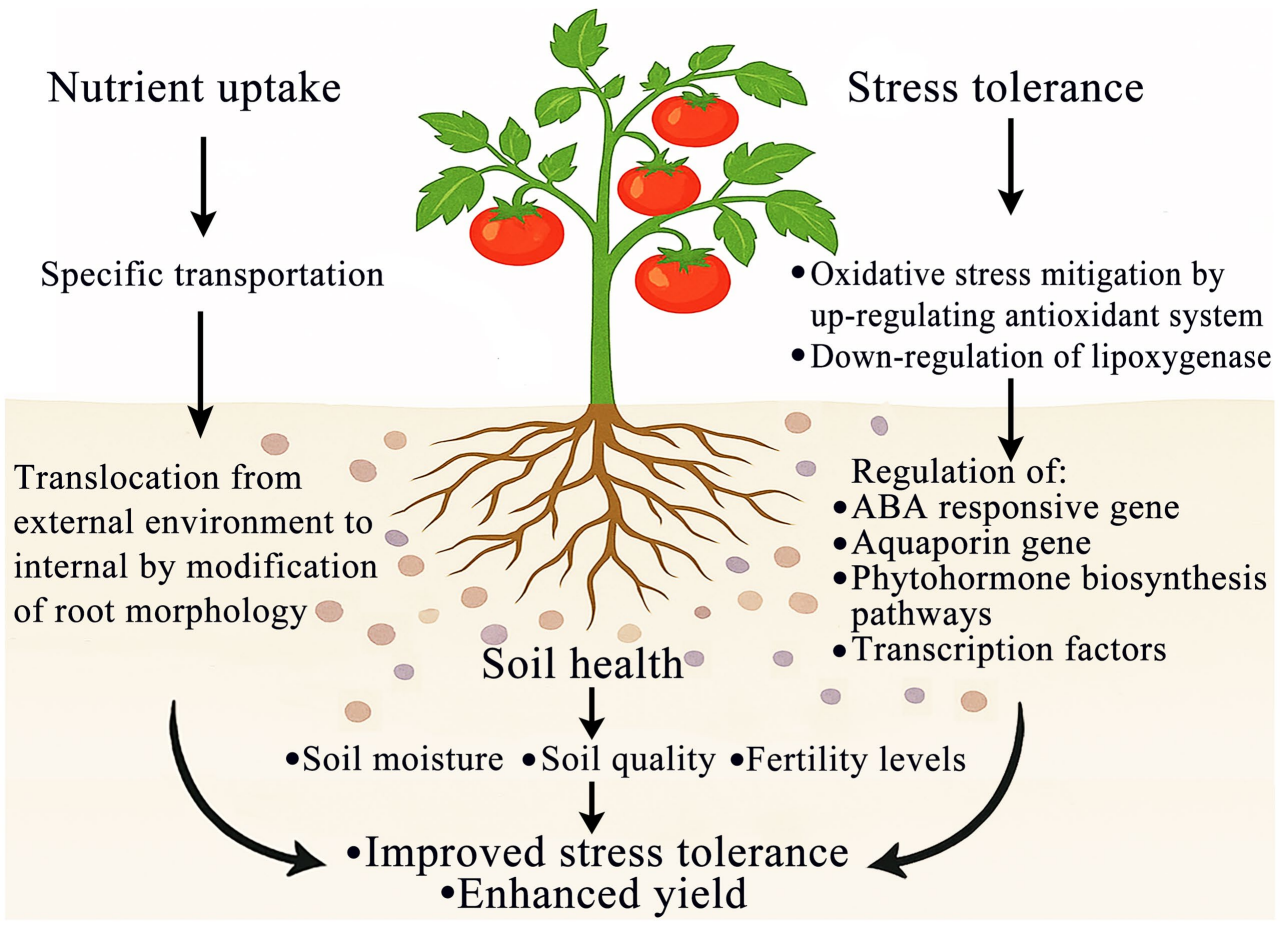
Schematic representation of the role of beneficial microorganisms in enhancing soil fertility and nutrient uptake by plants. Beneficial microorganisms improve soil health by enhancing soil moisture retention, quality, and fertility levels. These improvements facilitate nutrient uptake through specific transportation mechanisms and root morphology modification, enabling efficient translocation from the external environment to internal plant tissues. Concurrently, microorganisms bolster stress tolerance via upregulation of the antioxidant system and downregulation of lipoxygenase, thereby regulating ABA-responsive genes, aquaporin genes, phytohormone biosynthesis pathways, and transcription factors. The synergistic integration of these processes ultimately results in improved stress tolerance and enhanced crop yield.

Microbial inoculation supports plant survival in saline environments by enhancing the synthesis of osmoprotectants, such as glycine betaine and proline, and by activating antioxidant enzymes, including superoxide dismutase (SOD) and catalase (CAT). These responses help maintain osmotic balance and mitigate oxidative damage, thereby enhancing salt tolerance (Dimkpa et al., 2009). For instance, PGPR strains such as *Pseudomonas pseudoalcaligenes* and *Bacillus pumilus* have been shown to reduce lipid peroxidation, SOD and caspase-like activities, and programmed cell death in rice, thereby enhancing salinity tolerance in rice plants (Nonaka and Ezura, 2014; Jha and Subramanian, 2014; Bright et al., 2025). These PGPR strains also regulate the activities of antioxidant enzymes and stabilize cell membranes, further promoting plant growth under stress (Jha and Subramanian, 2014). Additionally, *Azospirillum brasilense* and various *Bacillus* spp. have been shown to support the production of osmolytes and suppress the biosynthesis of ethylene through the activity of 1-aminocyclopropane-1-carboxylic acid (ACC) deaminase. This enzymatic activity reduces the accumulation of reactive oxygen species (ROS) and strengthens the intrinsic antioxidant defense mechanism in plants (Nonaka and Ezura, 2014; Degon et al., 2023; Kaundal et al., 2025). Collectively, these observations underscore the critical role of plant–microbe interactions in regulating plant responses to abiotic stress. A deeper understanding of these mechanisms offers valuable opportunities to harness microbial strategies for enhancing plant resilience and productivity under adverse environmental conditions (Figure 1).

## 3 PGPR and tomato plants

**Figure 2:**
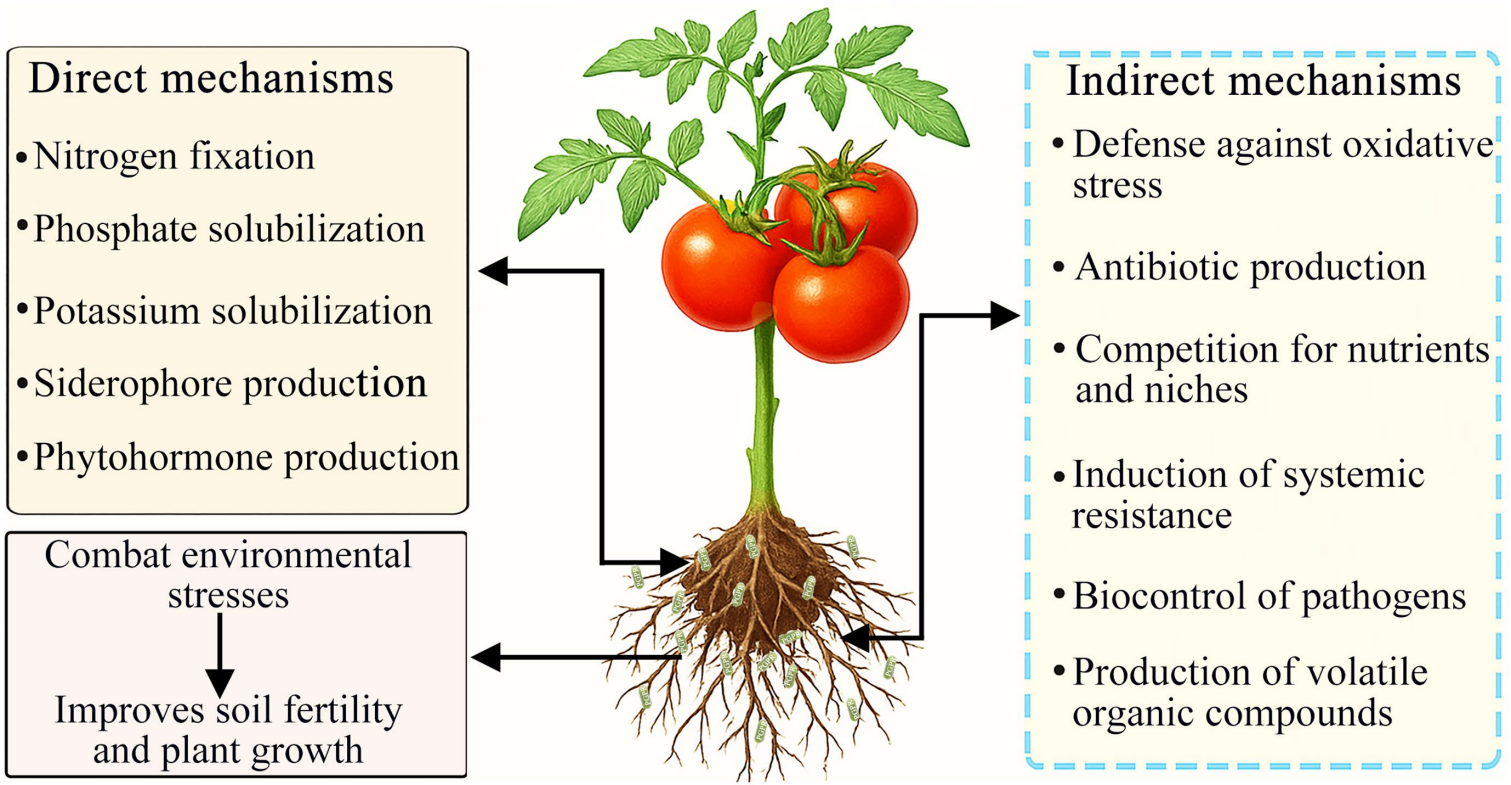
Diagram illustrating the direct and indirect mechanisms of plant growth promoting rhizobacteria (PGPR) in tomato plants.

PGPR are soil-dwelling bacteria that colonize plant roots and enhance plant growth (dos Santos et al., 2020; Nwachukwu et al., 2021). They improve plant health by directly modulating plant metabolism through their metabolic products, promoting root development, increasing enzyme activity, enhancing yield, and combating diseases (Fasusi et al., 2021; Zia et al., 2021). Additionally, these bacteria provide indirect protection by competing with phytopathogens for essential nutrients and space, producing antimicrobial compounds that inhibit pathogen germination, and inducing systemic defense responses in host plants (Basu et al., 2021). Furthermore, PGPR facilitate plant survival under abiotic stress conditions by enhancing fitness, increasing stress tolerance, and promoting phytoremediation potential (Brunetti et al., 2021; Ali et al., 2023). Current research aims to elucidate the rhizospheric bacterial systems that interact with plant roots and support their growth (Gómez-Godínez et al., 2023). The modifications induced by PGPR in plants are diverse, with growth promotion likely arising from a complex interplay among multiple pathways influencing both development and nutrition acquisition (Vocciante et al., 2022). PGPR exert beneficial effects on plant growth via direct and indirect mechanisms, with their impact on plant development representing a multifaceted process that varies depending on the specific bacterium and plant species involved (Mokrani et al., 2020) (Figure 2). The direct mechanisms underlying the enhancement of plant growth by PGPR include increased nutrient uptake and the modulation of plant hormone levels (Mohanty et al., 2021; Melini et al., 2023), whereas the indirect effects encompass a range of strategies for the prevention or management of plant diseases (Mekonnen and Kibret, 2021).

### 3.1 Roles of PGPR in promoting growth and nutrient utilization in tomato

Nitrogen is an abundant and essential macronutrient for plant growth and various metabolic processes (Govindasamy et al., 2023); however, its atmospheric form cannot be directly absorbed by plants and must be converted into ammonia (NH_3_) for utilization. PGPR mediate this conversion through biological nitrogen fixation (Klimasmith and Kent, 2022), with typical nitrogen-fixing PGPR species belonging to the genera *Azotobacter*, *Azospirillum*, *Burkholderia*, *Bradyrhizobium*, *Enterobacter*, *Gluconacetobacter*, and *Stenotrophomonas* (Singh et al., 2020). As the second most essential macronutrient, phosphorus is indispensable for photosynthesis, metabolism, and energy transfer in plants (Khan et al., 2023). PGPR can enhance the availability of soil phosphorus by mineralizing organic phosphorus and solubilizing insoluble inorganic phosphate—processes that are strongly influenced by soil pH (Cheng X. et al., 2023). These bacteria lower rhizospheric pH by secreting organic and inorganic acids, thereby enhancing soil phosphate bioavailability (Johan et al., 2021). Representative phosphate-solubilizing bacteria include members of the genera *Rhizobium*, *Pseudomonas*, and *Bacillus* (Kalayu, 2019).

Potassium, the seventh most abundant element in the Earth’s crust, is another essential macronutrient, following nitrogen and phosphorus (Dhillon et al., 2019; Olaniyan et al., 2022). Potassium participates in numerous metabolic and developmental processes and activates over 80 enzymes involved in starch synthesis, nitrate reduction, photosynthesis, and energy metabolism. An adequate supply of soil potassium has been shown to enhance plant vigor and increase resistance to biotic stresses, diseases, and pests (Srinivasarao et al., 2016). Efficient potassium-solubilizing strains include *Bacillus mucilaginosus*, *Bacillus edaphicus*, and *Bacillus circulans* (Umar et al., 2020; Kumar and Nautiyal, 2022). Iron is an essential micronutrient for all living organisms (Ferreira et al., 2019); however, its predominant naturally occurring form, Fe^3+^, exhibits low solubility and limited bioavailability (Timofeeva et al., 2022). Under conditions of iron limitation, PGPR secrete siderophores that chelate Fe^3+^, thereby enhancing iron uptake for plant growth while simultaneously competing with phytopathogens for iron and suppressing their proliferation (Hakim et al., 2021; Molnár et al., 2023; de Andrade et al., 2023).

Beyond nutrient acquisition, phytohormone production represents another key mechanism through which PGPR promote the growth of tomato plants (Kudoyarova et al., 2019). PGPR synthesize various phytohormones, including gibberellins, cytokinins, ABA, ethylene, auxins, and indole-3-acetic acid (IAA), which stimulate the proliferation of lateral roots, improve nutrient and water absorption, and support root cell development (Mukherjee et al., 2022; Sosnowski et al., 2023). Among these, IAA serves as a key phytohormone in plant–microbe interactions, whereas auxin production has been shown to modulate hormonal balance in plants, increase root biomass, and reduce stomatal size and density. In contrast, gibberellins and cytokinins promote shoot growth and facilitate root exudation (Backer et al., 2018; Lopes et al., 2021; Vocciante et al., 2022). The introduction of PGPR inoculants, including *Pseudomonas* and *Bacillus* spp., during tomato cultivation accelerates seed germination and enhances plant height, root length, leaf number, and fruit weight, often by improving phytohormone balance, nutrient availability, antimicrobial activity, and systemic resistance (Ketut Widnyana, 2019). Strains such as *Burkholderia contaminans* AY001 exhibit strong plant growth-promoting traits, including nitrogen fixation, phosphate solubilization, and IAA production, while also suppressing the growth of soil-borne pathogens and inducing systemic resistance in tomato plants (Heo et al., 2022). It has been demonstrated that the co-application of PGPR and AMF further enhances the uptake of potassium, activities of antioxidant enzymes, and accumulation of lycopene in tomato fruits, demonstrating synergistic effects on nutrient use efficiency and fruit quality (Ordookhani et al., 2010). Collectively, PGPR enhance the growth of tomato plants by improving nutrient availability, producing beneficial phytohormones, regulating root development, and supporting overall physiological vigor under diverse growth conditions.

### 3.2 PGPR as biocontrol agents and inducers of systemic resistance in tomato

Beyond their direct effects on promoting plant growth, PGPR significantly contribute to plant development by suppressing diseases and enhancing tolerance to biotic stressors (El-Saadony et al., 2022). This protective effect is primarily achieved through the microbial antagonism of pathogens and the stimulation of defense systems in the host plant. The resistance induced in plants typically arises from the activation of effective defense mechanisms, particularly systemic acquired resistance (SAR) and induced systemic resistance (ISR) (Bhadrecha and Bhawana, 2023). Signaling hormones, including salicylic acid (SA) and jasmonic acid (JA), serve as key regulators of these response pathways and represent core components of plant innate immunity (Yu et al., 2022).

In addition, PGPR can trigger ISR, with certain rhizobacterial strains conferring enhanced resistance against various pathogens, including *Fusarium oxysporum* and *Colletotrichum orbiculare* (Wei et al., 1991). Among PGPR, strains of *Pseudomonas*, *Bacillus*, and *Serratia* are the most extensively studied for their ability to elicit ISR (Meena et al., 2020). However, distinguishing ISR triggered by beneficial microbes from similar stress responses induced by pathogenic microorganisms remains challenging, as both activate analogous signaling pathways (Zhu et al., 2022). Numerous bacterial elicitors of ISR have been identified over recent decades, including quorum-sensing molecules, flagellin, acyl-homoserine lactones (AHLs), exopolysaccharides, lipopolysaccharides, and various secreted metabolites (Nag et al., 2021).

After perceiving non-pathogenic PGPR such as *Pseudomonas fluorescens* or *Bacillus subtilis*, tomato plants activate multiple layered defense mechanisms, including callose deposition, synthesis of phytoalexins, and upregulation of pathogenesis-related (PR) proteins (Yu et al., 2022; Joshi et al., 2023). These defense responses closely resemble those mounted during pathogenic infection. Additionally, PGPR reinforce physical defense barriers by modifying plant cell wall structure and promoting the deposition of lignin (Kaur et al., 2022).

Although functionally analogous to pathogen-induced SAR, the ISR mediated by beneficial bacteria distinctly relies on the NPR1 (nonexpressor of pathogenesis related genes 1) protein (Panpatte et al., 2020). In the SAR pathway, both NPR1 and TGA transcription factors (bZIP transcription factor) play an essential role in the transduction of SA signals, and their expression is further modulated by components of the MAPK (mitogen-activated protein kinase) signaling cascade (Han Q. et al., 2022; Zhao et al., 2024).

Inoculation with PGPR during tomato cultivation effectively reprograms defense metabolism, leading to the elevated accumulation of hydroxycinnamic acids, benzoic acid derivatives, flavonoids, and glycoalkaloids, thereby establishing a primed or “pre-stimulated” defensive state that enhances resistance to pathogens and herbivores (Mhlongo et al., 2020, 2022). For instance, *Klebsiella* sp. JCK-2201, which produces meso-2,3-butanediol, has been shown to alleviate bacterial wilt in tomato by upregulating defense-related genes and activating the SA and JA signaling pathways (Kim et al., 2022). Endophytic *Bacillus* sp. and *Pseudomonas aeruginosa* enhance resistance to cutworms by modulating the levels of IAA, SA, and ABA in tomato, while increasing the contents of phenolics and flavonoids and enhancing fruit yield (Kousar et al., 2020). *Bacillus subtilis* NSY50 promotes growth and photosynthetic efficiency in tomato while mitigating *Fusarium* wilt by regulating hormone signaling and the synthesis of secondary metabolites (Du et al., 2022). Similarly, *Burkholderia contaminans* AY001 displays multiple beneficial traits in plants and induces systemic resistance in tomato against *Fusarium oxysporum* f. sp. *lycopersici* and *Pseudomonas syringae* pv. (Heo et al., 2022). Collectively, PGPR enhance tolerance to biotic stresses in tomato through hormone modulation, defense priming, cell wall reinforcement, and the induction of systemic resistance, thus establishing these bacteria as sustainable and effective biocontrol agents in tomato production systems.

### 3.3 Role of PGPR in mitigating abiotic stress in tomato

PGPR act as eco-friendly biological stimulators that enhance the tolerance of tomato plants to various abiotic stresses, including drought, salinity, nutrient deficiency, and heavy metal toxicity, thereby supporting sustainable agricultural production (Numan et al., 2018). PGPR-mediated stress tolerance relies on coordinated physiological, biochemical, and molecular modifications, including improved nutrient acquisition, regulated phytohormone balance, enhanced antioxidant systems, stabilized osmotic equilibrium, optimized root system architecture, and reinforced root cell wall structures. Together, these integrated mechanisms alleviate stress-induced damage and help maintain normal growth and productivity in tomato under adverse environmental conditions (Figure 2).

The rhizosphere harbors abundant PGPR that form mutualistic interactions with the roots of tomato plants and promote plant growth through direct and indirect mechanisms (Martínez-Viveros et al., 2010; Garcia-Lemos et al., 2020). PGPR can directly promote plant growth through biological nitrogen fixation; solubilization of insoluble phosphorus, zinc, and potassium; and the synthesis of key phytohormones, including auxins, cytokinins, gibberellins, ethylene, and ABA (Großkinsky et al., 2016; Chauhan et al., 2021). These phytohormones regulate cell division, root elongation, development of lateral roots, and overall plant morphogenesis. Auxins, in particular, modify root architecture by promoting pericycle cell division, initiation of lateral root primordia, formation of root hairs, and primary root elongation, which markedly enhance water and nutrient uptake under conditions of drought and nutrient limitation (Tripathi et al., 2021; Roychoudhry and Kepinski, 2022). PGPR can indirectly promote plant growth and enhance stress adaptation in tomato through the biocontrol of soil-borne pathogens, production of siderophores, and the competitive exclusion of harmful microbes (Bal and Chanway, 2012; Glick, 2012). PGPR colonize root surfaces and internal tissues in tomato cultivation systems, facilitating nutrient uptake and enhancing tolerance to salinity, drought, and other environmental constraints (Jambon et al., 2018; Numan et al., 2018). When applied via seed priming or soil inoculation, PGPR strains such as *Pseudomonas* and *Bacillus* spp. accelerate seed germination, increase biomass accumulation, and improve fruit yield by enhancing nutrient availability, phytohormone production, and systemic stress resistance (Ketut Widnyana, 2019).

Abiotic stresses, including drought, salinity, and nutrient imbalances, induce the excessive accumulation of ROS in tomato plants, leading to oxidative damage, peroxidation of membrane lipids, protein denaturation, and disruption of cellular functions (Lanza and Reis, 2021; Zandi and Schnug, 2022). Under optimal conditions, plants maintain ROS homeostasis through intrinsic scavenging systems; however, exposure to severe stress frequently surpasses the capacity of these protective mechanisms (Hasanuzzaman et al., 2020). PGPR directly mitigate oxidative stress in plants by producing antioxidants and activating endogenous antioxidant defense pathways (Nivetha et al., 2021). The major enzymatic antioxidants induced by PGPR include SOD, CAT, and peroxidase (POD), which function sequentially to scavenge superoxide anions and hydrogen peroxide (H_2_O_2_), thereby mitigating oxidative stress (Jomova et al., 2024). PGPR also induce the accumulation of non-enzymatic antioxidants, including ascorbic acid and glutathione, which function synergistically in the ascorbate-glutathione cycle to efficiently scavenge ROS and maintain cellular redox balance (Zandi and Schnug, 2022; Mhamdi, 2023). PGPR modulate molecular signaling in their host plants to upregulate the expression of antioxidant genes, thereby conferring enhanced stress tolerance in tomato plants under harsh environmental conditions (Wahab et al., 2023a, 2023b).

Drought and salinity stress severely disrupt cellular osmotic balance in tomato plants, thereby impairing water uptake and inducing dehydration-related damage (Kumar et al., 2023). PGPR alleviate osmotic stress by promoting the synthesis and accumulation of compatible osmolytes, including proline and glycine betaine (Haghpanah et al., 2024). Proline functions as an effective osmoprotectant by maintaining cell turgor, stabilizing proteins and membranes, and directly scavenging ROS (Pattnaik et al., 2021). PGPR enhance the accumulation of proline by upregulating key biosynthetic genes, including Δ^1^-pyrroline-5-carboxylate synthetase (*P5CS*), under stress conditions (Hasanuzzaman et al., 2021). Similarly, glycine betaine maintains osmotic potential in the cytoplasm and vacuoles, thereby protecting tomato plants from drought and salinity-induced injuries (Rasheed et al., 2024). By stabilizing the osmotic equilibrium, PGPR enable tomato plants to retain water and sustain normal metabolic activities under abiotic stress conditions.

In addition, PGPR can significantly modify the architecture of tomato root systems by regulating root elongation, formation of lateral roots, proliferation of root hairs, and the spatial distribution of roots (Verma et al., 2022; Khan et al., 2021). These modifications are primarily mediated by PGPR-derived phytohormones, particularly through the dynamic balance between auxins and cytokinins (Pantoja-Guerra et al., 2023). Auxins promote the elongation of primary roots and the development of lateral roots, whereas cytokinins stimulate cell division and growth of root hairs, which collectively enhance the absorption area of roots (EL Sabagh et al., 2022; Wahab et al., 2025). PGPR strains, such as *Azospirillum brasiliense* and *Bacillus licheniformis*, enhance root growth in tomato plants via phytohormone production, thereby enhancing access to water and nutrients under conditions of drought and nutrient deficiency (Raja Gopalan et al., 2023). The optimized root system facilitates more efficient phosphate solubilization, nitrogen fixation, and siderophore-mediated iron acquisition, thereby further enhancing stress adaptation (Sagar et al., 2021; Hassen et al., 2023). Studies have demonstrated that PGPR-inoculated tomato plants consistently exhibit greater root length, density, and branching under conditions of abiotic stress compared to those of non-inoculated controls.

In addition to hormonal and metabolic regulation, PGPR enhance stress tolerance in tomato plants by remodeling the physicochemical properties of root cell walls (Vandana et al., 2021; Grover et al., 2021). The inoculation of PGPR induces the deposition of lignin and callose, which reinforce cell wall rigidity, enhance mechanical stability, and mitigate stress-induced damage (Choudhary et al., 2016; Chang et al., 2023). These cell wall modifications are key components of ISR, which not only confers resistance against biotic stresses but also contributes to abiotic stress tolerance (Sood et al., 2021; Wahab et al., 2025). PGPR-mediated cell wall fortification reduces cell wall plasticity and enhances the structural integrity of roots, thereby supporting continuous root growth under saline, drought, and heavy metal stress conditions (Srivastava et al., 2023).

In practical agricultural systems, PGPR effectively improve the performance of tomato plants under conditions of salinity, drought, nutrient limitation, and heavy metal stress. Halotolerant PGPR such as *Streptomyces* sp. KLBMPS084 enhance salt tolerance by increasing the activities of antioxidant enzymes, levels of osmoprotectants, and expression of stress-related genes (Gong et al., 2020). Under conditions of cadmium or chromium stress, PGPR reduce metal uptake, activate antioxidant systems, and promote plant growth by inducing the production of IAA and increasing phosphate solubilization (Gupta et al., 2020; Sarwar et al., 2022; Zhang et al., 2023; Zhou W. et al., 2022, 2024). The co-inoculation of PGPR with AMF has been shown to further enhance the content of lycopene, induce antioxidant activity, and increase the accumulation of potassium in tomato fruits (Ordookhani et al., 2010). Collectively, PGPR enhance the resilience of tomato plants to abiotic stresses through integrated and coordinated mechanisms, including phytohormone modulation, activation of antioxidant defense systems, osmotic adjustment, optimization of root architecture, reinforcement of cell wall integrity, and improved nutrient acquisition. These multifunctional benefits highlight the potential of PGPR as sustainable and effective biostimulants for enhancing the stability of tomato production under adverse environmental conditions.

## 4 AMF and growth of tomato plants

### 4.1 Symbiotic mechanisms of AMF in regulating tomato growth and stress resistance

**Figure 3:**
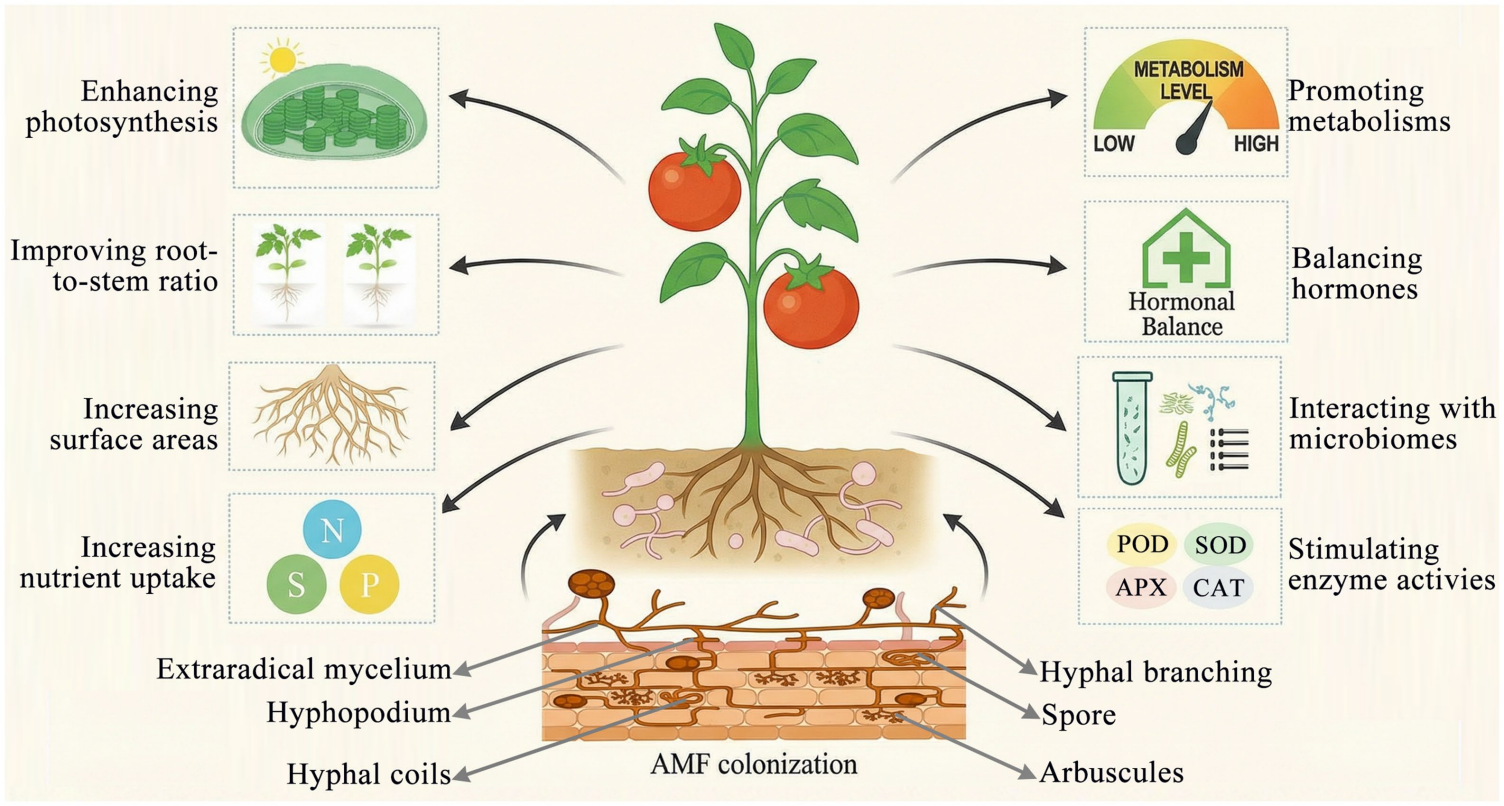
AMF enhance plant stress resilience through multiple synergistic pathways. These pathways include promoting photosynthetic efficiency, optimizing the root-to-stem ratio for improved resource allocation, expanding root surface area, and facilitating the uptake of nutrients such as nitrogen, phosphorus, and potassium via extraradical mycelium and arbuscular structures. Additionally, AMF boost metabolic processes, regulate hormonal balance—particularly involving abscisic acid and strigolactones—stimulate the activity of antioxidant enzymes (including superoxide dismutase, catalase, peroxidase, and ascorbate peroxidase), and interact with other beneficial microorganisms in the hyphosphere. Key structural features of AMF involved in these processes include spores, extraradical mycelium, hyphopodia, hyphal branching, hyphal coils, and arbuscules, which collectively support nutrient exchange, colonization, and the regulation of stress responses.

AMF, belonging to the phylum *Glomeromycota* are primarily classified into four orders, namely, *Glomerales*, *Archaeosporales*, *Paraglomerales*, and *Diversisporales*, which are further subdivided into 25 genera based on morphological features within the broader taxonomic framework (Redecker et al., 2013). AMF are obligate biotrophs that establish mutualistic associations with the majority of flowering plants (Bonfante and Genre, 2010). In plant–AMF symbiotic associations, AMF connect plant roots to the surrounding soil via intraradical hyphae (IRH), which penetrate the outer cortical cells of plant roots and form hyphal coils enclosed by a plant-derived plasma membrane (Genre et al., 2020). Within the inner cortical cells, AMF form arbuscules that are surrounded by plant-derived perifungal membranes, thereby establishing the peri-arbuscular space (PAS). This compartment serves as the site for bidirectional nutrient exchange between the AMF and the host plant (Wipf et al., 2019; Duan et al., 2024). Colonization by AMF significantly enhances nutrient absorption, increases stress tolerance, and alters the composition of the rhizospheric microbiome by recruiting other beneficial microorganisms. This symbiotic interaction plays a critical role in shaping the microbial ecology of the rhizosphere and supporting overall plant health (Cheng Y. et al., 2023; Boyno et al., 2025). AMF contribute to enhanced plant growth, improved mineral nutrition, and increased resilience against abiotic stresses, including drought, salinity, and heavy metal toxicity, as well as biotic stresses such as soil-borne diseases (Figure 3) (Lenoir et al., 2016; Brar et al., 2024; Sun et al., 2024). AMF enhance the performance of tomato plants during cultivation by promoting the uptake of phosphorus and nitrogen, modulating hormonal signaling, enhancing the activities of antioxidant enzymes, and improving WUE. These benefits contribute to higher yield and fruit quality, highlighting AMF as a promising biotechnological tool for promoting sustainable agriculture (Chandrasekaran et al., 2021).

### 4.2 AMF-mediated biocontrol and disease resistance in tomato

Numerous studies have confirmed that AMF enhance plant resistance to a wide range of pathogens (Umer et al., 2025). AMF have also been shown to interact synergistically with various rhizospheric microbes, including those in the genera *Trichoderma*, *Pseudomonas*, and *Bacillus*, thereby improving collective biocontrol efficacy against pathogenic bacteria, fungi, and nematodes (Umer et al., 2025). This synergy is achieved through the enhanced secretion of antimicrobial compounds, activation of plant defense pathways, and modification of the rhizospheric microbiome, thereby creating an unfavorable environment for plant pathogens (Umer et al., 2025; Farhaoui et al., 2025). AMF have been shown to effectively suppress soil-borne fungal pathogens responsible for root rot and wilt diseases in various crops, as well as foliar pathogens such as *Alternaria solani*, which affects tomato plants (Harrier and Watson, 2004).

Mycorrhizal symbiosis confers broad-spectrum protection against viruses, bacteria, phytoplasmas, fungi, and herbivorous pests through three interconnected mechanisms (Comby et al., 2017). First, AMF enhance photosynthetic efficiency and the acquisition of nutrients, particularly phosphorus and nitrogen, thereby strengthening host vigor and increasing resistance to pathogen colonization and proliferation (van der Heijden et al., 2006; Hildebrandt et al., 2007). Second, the extraradical mycelium (ERM) of AMF modulates the rhizospheric microbiome by competing spatially and nutritionally with soil-borne pathogens, while simultaneously stimulating the activity of PGPR, including nitrogen-fixing and phosphate-solubilizing bacteria, which further suppress pathogenic microorganisms (Schouteden et al., 2015; Pérez-de-Luque et al., 2017; Nacoon et al., 2020). Third, AMF establish common mycorrhizal networks (CMNs) among conspecific or heterospecific plants, thereby facilitating long-distance nutrient translocation and interplant signal transfer, including the induction of membrane depolarization (Babikova et al., 2013; Johnson and Gilbert, 2015; Bücking et al., 2016). These CMNs facilitate the transmission of defense signals from AMF-colonized plants under attack by caterpillars or necrotrophic fungi to neighboring plants, thereby triggering the preemptive activation of plant defense responses (Babikova et al., 2013, 2014; Song et al., 2010, 2015).

The establishment of plant–AMF symbiosis initiates a tightly regulated immune response cascade in the host plant. During the early stages of the symbiotic interaction, plant pattern recognition receptors (PRRs) recognize AMF-secreted microbe-associated molecular patterns (MAMPs), thereby triggering local MAMP-triggered immunity (MTI) in roots and concomitant SA biosynthesis (Zhang and Zhou, 2010; Zamioudis and Pieterse, 2012). SA-derived long-distance signals systemically prime SA-dependent defenses in aerial tissues; however, elevated SA levels in roots inhibit the colonization of AMF (De Román et al., 2011; Cameron et al., 2013). In response, AMF secrete effector proteins that induce the production of ABA in roots, leading to the local suppression of SA-mediated defense mechanisms and successful root colonization. Additionally, ABA translocates to aerial tissues via the xylem, thereby reinforcing cell wall defenses against foliar pathogens (Ton et al., 2009; Trouvelot et al., 2015). Once symbiosis is established, AMF induce systemic mycorrhiza-induced resistance (MIR) in the distal tissues of the host plant (Jung et al., 2012). Cordier et al. confirmed this phenotype using a split-root system, in which tomato roots colonized by *Funneliformis mosseae* (syn. *Glomus mosseae*) conferred resistance to *Phytophthora nicotianae* var. *parasitica* in non-mycorrhizal roots, thereby reducing necrosis and the growth of pathogen mycelia via the accumulation of non-esterified pectins and PR1a proteins in the cell wall (Delaeter et al., 2024). MIR is predominantly governed by hormonal signaling networks, and although it may occasionally resemble SA-dependent SAR, it more frequently aligns with JA/ethylene-dependent ISR (Trouvelot et al., 2015). Furthermore, AMF modulate MIR by enhancing phosphate uptake by plant roots and the translocation of photosynthates to the roots, thereby altering the composition of root exudates to recruit beneficial rhizobacteria, including *Pseudomonas* and *Burkholderia* spp., which secrete signaling compounds that amplify JA/ethylene-dependent ISR via long-distance systemic priming (Liu et al., 2007; Van der Ent et al., 2009; Smith and Smith, 2011; Jung et al., 2012; Pieterse et al., 2009, 2014).

The hormonal reprogramming underlying MIR drives pathogen-specific defense responses. JA- and ethylene-dependent pathways predominantly confer resistance against necrotrophic organisms and chewing insects, and, to a lesser extent, hemibiotrophic pathogens (Heidel and Baldwin, 2004; Pozo and Azcón-Aguilar, 2007; Pieterse et al., 2014). These pathways are characterized by the upregulation of lipoxygenase D gene (*LOXD*) and allene oxide cyclase gene (*AOC*); increased activities of polyphenol oxidase (PPO), phenylalanine ammonia lyase (PAL), and β-1,3-glucanase; and the accumulation of phenolic compounds, PR1a, callose, and pectin at the sites of pathogen penetration (Jaiti et al., 2008; Wang et al., 2022). In contrast, SA-dependent defense responses, which are primarily effective against hemibiotrophic and biotrophic pathogens, are characterized by the accumulation of pathogenesis-related (PR) proteins, production of ROS, reinforcement of the cell wall via the enhanced deposition of callose and phenolic compounds, and activation of the phenylpropanoid pathway (Volpin et al., 1994; Fester and Hause, 2005; Song et al., 2015). Field and greenhouse studies have confirmed that AMF suppress both soil-borne and foliar pathogens in tomato plants, and exert synergistic effects when combined with rhizospheric microbes such as *Trichoderma*, *Pseudomonas*, and *Bacillus* spp. (Harrier and Watson, 2004; Umer et al., 2025). This suppressive effect is attributed to the enhanced secretion of antimicrobial compounds, coordinated activation of defense pathways, and restructuring of the rhizospheric microbiome, which collectively create a pathogen-suppressive environment (Farhaoui et al., 2025; Umer et al., 2025). Specifically, *Funneliformis mosseae* induces systemic resistance in tomatoes, reducing infections caused by *Meloidogyne incognita* (root-knot nematode) and *Pratylenchus penetrans* (root-lesion nematode) by 45 and 87%, respectively, even when the AMF and nematodes colonize spatially distinct root zones. This effect is mediated via the activation of the JA pathway and enhanced root structure and biochemical resistance (Vos et al., 2012). Furthermore, *Rhizophagus intraradices* (syn. *Glomus intraradices*) alleviates damage to plant roots caused by *Nacobbus aberrans* (false root-knot nematode) by reducing gall formation and suppressing nematode reproduction, with maximal efficacy observed under preventive inoculation regimes (Lax et al., 2011). Additionally, inoculation with AMF mitigates bacterial wilt caused by *Ralstonia solanacearum* by 65.7% through modulation of soil pH, enhancement of root phenolic content, and activation of leaf defense-related enzymes, including PPO and POD, thereby limiting yield loss during tomato fruit maturation (Li et al., 2021). AMF also prime JA-dependent defense responses, thereby reducing the severity of *Alternaria solani*, which causes early blight in tomato plants. AMF-primed plants exhibit the rapid activation of PAL, lipoxygenase (LOX), chitinase, and β-1,3-glucanase, along with the upregulation of *PR* genes, including *PR1*, *PR2*, and *PR3*, upon pathogen challenge (Song et al., 2015). Furthermore, *Rhizophagus irregularis* has been shown to mitigate damage caused by *Botrytis cinerea*, the causative agent of gray mold in tomato, through ISR, demonstrating sustained biocontrol efficacy when integrated into microbial consortia (Minchev et al., 2021). Additionally, *Glomus* spp. suppress *Fusarium oxysporum* f. sp. *lycopersici*, the causal agent of Fusarium wilt, while enhancing the growth of tomato plants; uptake of nutrients, including nitrogen, phosphorus, and potassium; chlorophyll content; and fruit yield under pot culture conditions (Kumari and Prabina, 2019; Kumari et al., 2019).

Collectively, these multifaceted mechanisms—direct pathogen competition, induction of systemic defense, and the promotion of beneficial soil–plant interactions—position AMF as a sustainable and eco-friendly alternative to synthetic phytosanitary products for the management of diverse pests and pathogens in tomato cultivation. However, the translational efficacy of the benefits observed in the laboratory is tempered by AMF-specific and environmental factors under field conditions.

### 4.3 Synergistic mechanisms underlying AMF-mediated alleviation of drought stress in tomato

Drought is a significant abiotic stressor that severely inhibits the growth of tomato plants by disrupting nutrient absorption, diminishing physiological efficiency, and ultimately reducing yield. To mitigate these adverse effects, integrated management strategies are essential, with AMF representing a promising biological approach. AMF exert multifaceted regulatory effects on tomato plants, effectively alleviating drought stress through the coordinated modulation of physiological metabolism, molecular regulation, nutrient uptake, and hormonal balance (Augé, 2001; Augé et al., 2015). Different species and strains of AMF exhibit species-specific regulatory characteristics, with ABA serving as a key signaling molecule in mediating the symbiotic relationship between AMF and host plants and playing a central role in regulating stress resistance (Herrera-Medina et al., 2007; Martín-Rodríguez et al., 2010; Xu et al., 2018). Specifically, plant–AMF symbiosis mitigates drought stress in tomato plants by reshaping the molecular expression profile, optimizing physiological responses, and regulating hormonal homeostasis, with these responses closely associated with the ABA synthesis capacity and AMF species specificity of the host (Chitarra et al., 2016; Xu et al., 2018).

Inoculation with AMF upregulates the expression of 14–3-3 genes *TFT2* and *TFT3*, thereby reducing transpiration and enhancing WUE in wild-type tomato under drought conditions. In contrast, AMF specifically induce the expression of *TFT5*, *TFT7*, *TFT9*, and *TFT10* in ABA-deficient mutants, thereby modulating transpiration responses to mitigate the decline in WUE (Xu et al., 2018). Moreover, ABA plays an essential role in the establishment and maintenance of functional plant–AMF symbiosis, as evidenced by the dependence of AMF colonization and symbiotic performance on the ABA biosynthetic capacity of the host plant (Aroca et al., 2008; Herrera-Medina et al., 2007; Martín-Rodríguez et al., 2010; Xu et al., 2018). Notably, ABA plays critical roles in both the initiation and maintenance of plant–AMF symbiosis, which in turn promotes the development and functional activity of the fungal symbiont (Aroca et al., 2008; Herrera-Medina et al., 2007; Martín-Rodríguez et al., 2010). For instance, various species of AMF, including *Funneliformis mosseae* and *Rhizophagus intraradices*, enhance drought tolerance in tomato by regulating Gs, modulating ABA content, and enhancing the activities of antioxidant enzymes (Chitarra et al., 2016), thereby further illustrating the synergistic interplay between AMF and ABA in mediating drought resistance.

Previous studies have demonstrated that AMF establish a stress-resistant regulatory network in which ABA, strigolactone, and JA serve as the core components (Chitarra et al., 2016; Ruiz-Lozano et al., 2016; Xu et al., 2015). These hormones act in concert to mediate plant–AMF symbiosis and enhance drought resistance in tomato. Under drought stress, tomato plants engaged in AMF symbiosis exhibit upregulation of the strigolactone synthesis gene *SlCCD7*, while *SlCCD8* expression remains unchanged, resulting in elevated strigolactone levels that facilitate plant–AMF symbiosis. Root colonization by AMF exhibits a strong correlation with elevated strigolactone levels and the severity of drought stress (Ruiz-Lozano et al., 2016). Further research is necessary for confirming the intrinsic relationship between strigolactone biosynthesis and plant–AMF symbiosis under drought conditions. Colonization by AMF also upregulates the JA synthesis gene *SlLOXD* under both normal and drought conditions, contributing to the stabilization of the symbiotic relationship. Meanwhile, colonization by *Septoglomus constrictum* downregulates the ABA synthetic gene *SlNCED* under drought conditions, thereby maintaining higher Gs and plant water status. This coordinated regulation, involving the simultaneous enhancement of JA levels and suppression of ABA synthesis, contributes to maintaining the balance between symbiotic stability and plant water retention (Xu et al., 2015). Additionally, certain AMF species increase the levels of ABA glucosyl ester (ABA-GE) in roots, which serve as a reserve pool for rapid production of active ABA under stress and further reinforce the central regulatory role of ABA in the AMF–tomato symbiotic drought-resistance system (Chitarra et al., 2016; Rivero et al., 2018).

AMF play a crucial role in activating the plant antioxidant system, thereby mitigating drought-induced oxidative damage—a key physiological mechanism for protecting the symbiotic system and maintaining normal plant metabolism. Specifically, *Funneliformis mosseae* and *Rhizophagus intraradices* enhance the activity of key antioxidant enzymes, including SOD, ascorbate peroxidase (APX), and POD (Chitarra et al., 2016). Additionally, *Septoglomus constrictum* reduces lipid peroxidation and the accumulation of hydrogen peroxide (H_2_O_2_) under drought stress, while simultaneously increasing the activities of antioxidant enzymes (Duc et al., 2018). Furthermore, three glutathione S-transferases (GSTs) in *Rhizophagus intraradices* are upregulated under drought conditions, thereby enhancing oxidative stress defense mechanisms in both the fungal symbiont and its host plant. This regulation is essential for ensuring the proper development of arbuscules and the maintenance of functional nutrient transport (Balestrini et al., 2019; Xu et al., 2015).

AMF modulate Gs and coordinately regulate the expression of plant and fungal aquaporin (*AQP*) genes, thereby enhancing root hydraulic conductivity. This not only enhances the water absorption capacity of the host plant but also establishes a foundation for maintaining stable photosynthetic efficiency (Davies Jr et al., 1996; Augé et al., 2015; Chitarra et al., 2016). Under drought conditions, AMF upregulate specific plant *AQP* genes, such as *LeNIP3;1*, and fungal *AQP* genes, including *RiAQPF2*, while also modulating the expression of other genes, such as *LePIP1;1* and *LeTIP2;3* (Chitarra et al., 2016). The external hyphae of AMF can penetrate soil pores that are inaccessible to plant roots, thereby expanding the water absorption range and enhancing root hydraulic conductivity. The enhanced development of extraradical hyphae is crucial for mitigating drought stress in tomato plants (Davies Jr et al., 1996; Augé et al., 2015). Notably, the enhancement of root hydraulic conductivity facilitates the transmission of hydraulic signals from the roots to the above-ground tissues of the host plant, as evidenced by the maintenance of high Gs levels (Rodríguez et al., 1997).

Colonization by AMF significantly enhances the growth and nutrient acquisition efficiency of tomato plants under drought stress. The growth-promoting effects of AMF are species-specific and are closely associated with the regulation of carbohydrate allocation and nutrient transport genes (Dell'Amico et al., 2002; Berruti et al., 2013; Rivero et al., 2015; Duc et al., 2018; Xu et al., 2018). Specifically, AMF substantially increase the dry biomass, shoot biomass, and leaf surface area of drought-stressed tomato seedlings. For instance, *Glomus clarum* is particularly effective in promoting shoot biomass accumulation by facilitating the preferential allocation of a greater proportion of carbohydrates to the shoots (Dell'Amico et al., 2002; Rivero et al., 2015). In contrast, inoculation with *Septoglomus constrictum* increases the root and shoot dry weight of tomato plants by 14–18% compared to that of non-colonized plants under water stress (Duc et al., 2018). Furthermore, wild-type tomato plants exhibit a higher mycorrhizal colonization rate than mutant varieties under drought stress (Xu et al., 2018). Both single and mixed AMF inocula exhibit varying regulatory effects on tomato traits, whereas indigenous AMF isolates generally exert more pronounced growth-promoting effects, likely due to their adaptation to local environmental conditions (Berruti et al., 2013, 2016; Mannino et al., 2020).

The enhancement of nutrient acquisition efficiency by AMF further synergistically improves drought resistance in tomato, as the adequate availability of nutrients is crucial for maintaining plant physiological functions under stress. AMF significantly increase the uptake of nitrogen, phosphorus, calcium, magnesium, iron, and other essential nutrients in drought-stressed tomato plants (Subramanian et al., 2006; Chitarra et al., 2016; Volpe et al., 2018; Xu et al., 2018). *Rhizophagus intraradices* increases shoot phosphorus concentration in wild-type tomato plants by 23% under drought conditions, compared to a 14% increase under well-watered conditions (Xu et al., 2018). *Rhizophagus intraradices* also optimizes nitrogen uptake by modulating the mobility of nitrate (NO_3_^−^) ions under conditions of water deficiency (Subramanian et al., 2006). The symbiotic inorganic phosphate (Pi) uptake pathway is central to AMF-mediated phosphorus transport (Bucher, 2007; Javot et al., 2007; Smith and Smith, 2011), with eight genes in the PHT1 family mediating Pi uptake by tomato roots (Chen et al., 2014). These *PHT1* genes are induced by mycorrhizal colonization (Nagy et al., 2005, 2009). The *LePT4* and *LePT5* genes are upregulated under drought stress, exhibiting the highest expression levels in plants colonized by *Funneliformis mosseae*. Conversely, *LePT3* does not participate in the drought stress response, whereas *LePT1* and *LePT2* exhibit opposing expression patterns. *LePT2* and *LePT4* play significant roles in enhancing drought tolerance by promoting the absorption of Pi and contributing to the synthesis of photosynthetic pigments and antioxidant enzymes (Volpe et al., 2018).

In response to drought conditions, the cytochrome P450 genes are upregulated in tomato roots colonized by *Rhizophagus intraradices*, thereby facilitating sterol synthesis, which is essential for arbuscule formation and supports the proper development of the symbiotic system and nutrient transport functions (Handa et al., 2015; Balestrini et al., 2019). Additionally, the fungal genes associated with the ‘conidiation protein 6’ domain and signal transduction-related domains in *Rhizophagus intraradices* are upregulated under drought stress. Fungal conidiation can be induced by nutrient deprivation or the desiccation of mycelia, aiding AMF in adapting to drought stress, maintaining symbiotic stability with tomato plants, and sustaining regulatory effects (Balestrini et al., 2019).

AMF symbiosis enhances the photosynthetic efficiency of tomato plants under stress by optimizing the photosynthetic apparatus, gas exchange parameters, and WUE, thereby providing a direct physiological basis for mitigating stress-induced reductions in yield (Dell'Amico et al., 2002; Subramanian et al., 2006; Chitarra et al., 2016; Ruiz-Lozano et al., 2016). Furthermore, this process is closely linked to enhanced water and nutrient availability mediated by AMF. Colonization by AMF preserves the normal functioning of photosystem II (PS II) during drought stress, with *Septoglomus constrictum* significantly increasing the Fv/Fm ratio in tomato plants (Duc et al., 2018). Plants inoculated with AMF exhibit no significant decline in photosynthetic rate (Pn) under drought conditions, contrasting sharply with the marked reductions observed in non-inoculated plants (Xu et al., 2018). Under field conditions, AMF-inoculated tomatoes maintain higher leaf relative water content (RWC), which provides a robust foundation for stable photosynthesis (Subramanian et al., 2006). As aforementioned, AMF enhances Gs in tomato plants under drought stress, with *Septoglomus constrictum* increasing Gs by 200% (Duc et al., 2018). This enhancement increases intracellular CO_2_ concentration and promotes photosynthetic activity (Dell'Amico et al., 2002). The Pn and transpiration rate (Tr) of AMF-inoculated plants are adaptively regulated under stress, with *Rhizophagus intraradices* increasing Tr in ABA mutant plants under well-watered conditions and improving WUE in wild-type plants under drought stress (Xu et al., 2018).

AMF significantly reduce transpiration loss and optimize water allocation in wild-type tomato plants, while modulating transpiration responses in ABA-deficient mutants to maintain WUE (Xu et al., 2018). The enhanced nutritional status, particularly phosphorus accumulation, together with improved water status in colonized plants, synergistically contribute to increased WUE. This synergistic effect is the primary reason underlying the consistently higher fruit yield in AMF-inoculated tomato plants across varying drought intensities (Chitarra et al., 2016). Furthermore, the increased Gs and root hydraulic conductivity observed in AMF-colonized plants correlate with enhanced water uptake by the roots. Notably, the recovery of non-inoculated plants under fully watered conditions does not restore the Pn and Gs, thereby confirming that AMF exert a stable and long-term regulatory effect on the photosynthetic system of tomato plants (Dell'Amico et al., 2002).

### 4.4 AMF-mediated amelioration of salt stress in tomato plants

Soil salinization is a significant agricultural and eco-environmental challenge, particularly in arid and semi-arid regions worldwide, where it severely limits plant growth. Salinity reduces soil water potential and promotes the accumulation of toxic ions such as Na^+^ and Cl^−^, leading to water deficits, nutritional imbalances, and ion toxicity in plants, thereby disrupting nutrient uptake, osmotic regulation, and cellular functions (van Zelm et al., 2020). High salinity critically impairs seed germination, vegetative growth, fruit yield, and overall quality in tomato plants (Yurtseven et al., 2005; Roșca et al., 2023). AMF are obligate biotrophs belonging to the phylum *Glomeromycota* and establish mutualistic symbioses with most flowering plants. AMF–plant symbiosis has emerged as an environmentally friendly strategy to mitigate salt stress in tomato plants, particularly when combined with compost application (Hajiboland et al., 2010; Abdel Latef and Chaoxing, 2011a; Mekkaoui et al., 2024).

AMF mitigate salt stress through various interconnected mechanisms, beginning with their role in promoting growth and nutrient uptake in tomato plants. Salt stress markedly diminishes dry matter accumulation in the roots, stems, and leaves, as well as leaf area and the fresh and dry weights of shoots and roots, primarily due to osmotic stress and ion-specific toxicity from Na^+^ and Cl^−^ (Hajiboland et al., 2010; Tanveer et al., 2020). Inoculation with AMF effectively counteracts these growth inhibitions, exerting a more pronounced positive effect on above-ground biomass, attributable to the preferential allocation of carbohydrates toward root colonization (Abdel Latef and Chaoxing, 2011b). For instance, colonization by *Funneliformis mosseae* has been shown to enhance dry matter content and leaf area, while *Rhizophagus intraradices* is particularly beneficial for salt-tolerant cultivars such as Piazar, compared with salt-sensitive varieties like Behta (Hajiboland et al., 2010; Abdel Latef and Chaoxing, 2011a). AMF-inoculated seedlings exhibit a higher accumulation of dry matter in saline soils (0.098 g plant^−1^) compared to that in non-inoculated counterparts (0.082 g plant^−1^) (Balliu et al., 2015). Through extensive extraradical hyphal networks, AMF expand nutrient absorption ranges, enhancing the uptake of nitrogen, phosphorus, potassium, calcium, magnesium, iron, manganese, and zinc, while simultaneously reducing Na^+^ accumulation in fruits (Ebrahim and Saleem, 2017; Kong et al., 2020). Phosphorus nutrition is particularly crucial, as AMF facilitate phosphorus uptake via vacuolar membrane integration and Na^+^ compartmentalization, even under low-phosphorus conditions (Cantrell and Linderman, 2001). Furthermore, AMF promote the accumulation of osmotic regulators, including soluble sugars, proline, betaine, and polyamines, which help maintain cellular osmotic balance in salt-stressed plants (Evelin et al., 2009; Kong et al., 2020). Additionally, co-inoculation with *Rhizophagus clarum* and *Rhizophagus intraradices* enhances fruit quality by increasing the contents of vitamin C, soluble sugar, and lycopene, as well as single-fruit weight and yield per plant (Evelin et al., 2009; Kong et al., 2020; Chandrasekaran et al., 2021).

Another key mechanism for maintaining ionic homeostasis is the regulation of ion balance, which is crucial for plant growth under salt stress. Ion imbalance, resulting from competition between Na^+^/Cl^−^ and essential nutrients, significantly constrains the growth of tomato plants (Dasgan et al., 2002). AMF play a vital role in regulating ion uptake and transport, with the K^+^/Na^+^ ratio serving as a key indicator of salt tolerance (Hajiboland et al., 2010). AMF restrict the translocation of Na^+^ from the roots to shoots, thereby increasing the K^+^/Na^+^, Ca^2+^/Na^+^, and Mg^2+^/Na^+^ ratios in the leaves and stems, which protects the photosynthetic organs (Hajiboland et al., 2010; Santander et al., 2019). Mycorrhizal plants, particularly those colonized by *Funneliformis mosseae*, consistently exhibit higher K^+^ and lower Na^+^ levels across varying salinity gradients (Abdel Latef and Chaoxing, 2011a). Furthermore, salt-adapted *Rhizophagus etunicatum* has been shown to be more effective in reducing leaf Na^+^ levels compared to *Funneliformis mosseae* and *Rhizoglomus irregulare* (Rivero et al., 2018). A high K^+^/Na^+^ ratio is essential for maintaining cytoplasmic balance, promoting Na^+^ efflux, and preventing disruptions in metabolic processes and protein synthesis (Hajiboland et al., 2010; Augé et al., 2015). AMF also regulate the expression of *AQP* genes such as *GintAQP1* in *Rhizophagus intraradices*, as well as Na^+^/H^+^ antiporter genes such as *LeNHX1* and *LeNHX2*, to maintain ionic homeostasis (Ouziad et al., 2006).

Inoculation with AMF has been shown to enhance the photosynthetic capacity and water status in salt-stressed tomatoes (Chandrasekaran et al., 2021). Salt stress induces stomatal closure, reduces the chlorophyll content, and inhibits the assimilation of CO_2_, thereby impairing the photosynthetic efficiency (Lin et al., 2017). However, inoculation with AMF mitigates these adverse effects by enhancing the concentration of chlorophyll, net Pn, Gs, and Tr, while simultaneously promoting the assimilation of CO_2_ (Abdel Latef and Chaoxing, 2011a; Chandrasekaran et al., 2019). For instance, plants inoculated with *Rhizophagus intraradices* exhibit higher Tr and Gs, whereas *Rhizophagus fasciculatus* enhances chlorophyll synthesis by increasing leaf nitrogen and magnesium content and reducing sodium uptake (Ebrahim and Saleem, 2017; Kong et al., 2020). An elevated chlorophyll a/b ratio indicates enhanced light-harvesting efficiency and improved salt adaptation (Ebrahim and Saleem, 2017). Inoculation with AMF also increases root hydraulic conductivity and improves water absorption through hyphal networks that penetrate soil pores inaccessible to plant roots (Augé et al., 2015; Ruiz-Lozano et al., 2016). Although inoculation with AMF may reduce WUE owing to increased transpiration, improvements in water uptake and photosynthetic performance collectively contribute to enhanced plant growth (Kong et al., 2020). Additionally, the AMF-induced accumulation of JA in roots—particularly in *Rhizophagus etunicatum*-colonized plants—serves as a bio-protector, while metabolites such ABA-GE and β-ionone further regulate water balance and stress responses (Rivero et al., 2018).

AMF enhance the activity of the antioxidant system, thereby alleviating oxidative stress. Salt stress induces the production of ROS, including superoxide radicals (O_2_^−^), H_2_O_2_, and hydroxyl radicals (OH^−^), which can damage proteins, nucleic acids, and lipids, ultimately disrupting membrane integrity (Apel and Hirt, 2004). Inoculation with AMF enhances the activity of ROS-scavenging enzymes, including SOD, POD, APX, and CAT (He et al., 2007; Abdel Latef and Chaoxing, 2011a). For instance, tomatoes inoculated with *Funneliformis mosseae* exhibit increased enzyme activities (except for SOD and CAT at 100 mM NaCl) and reduced levels of H_2_O_2_ and malondialdehyde (MDA), both of which serve as indicators of reduced lipid peroxidation and membrane damage (Abdel Latef and Chaoxing, 2011a). Furthermore, AMF colonization promotes the accumulation of proline, a non-enzymatic antioxidant that scavenges ROS and stabilizes cellular structures (Hajiboland et al., 2010). The lower MDA content and reduced membrane leakage observed in inoculated plants further corroborate the protective role of the antioxidant system (Al-Karaki et al., 2001). Collectively, these mechanisms position AMF as a promising biostimulant for enhancing tomato productivity in saline soils, thereby contributing to more resilient and sustainable agricultural systems.

## 5 Synergistic interactions between AMF and PGPR: mechanisms and agronomic implications

### 5.1 Mechanistic foundations of AMF-PGPR co-inoculation

**Figure 4:**
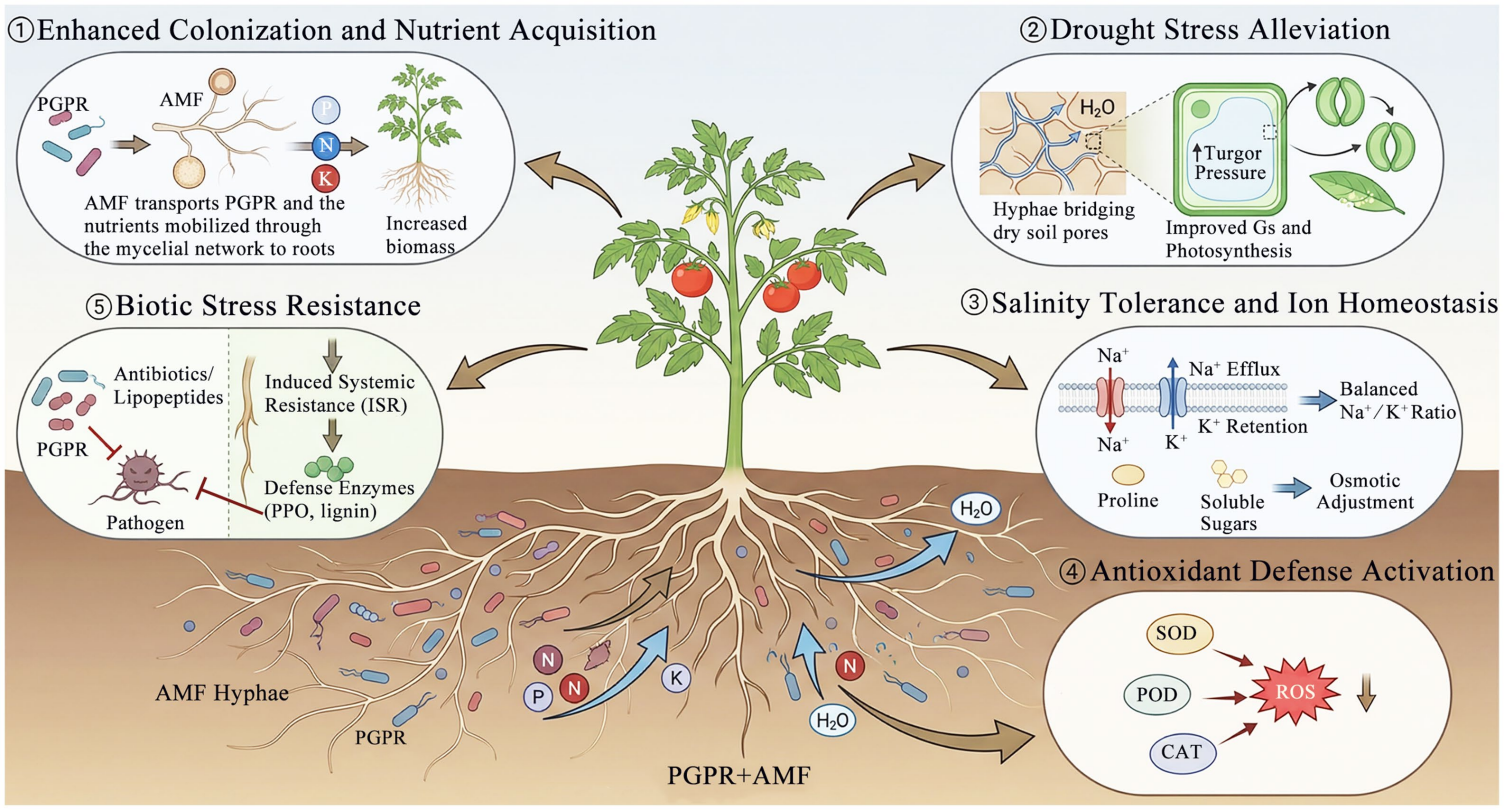
Synergistic mechanisms of PGPR and AMF in enhancing tomato growth and stress resilience. (1) PGPR increases the availability of soil nutrients, while AMF facilitates the transport of PGPR and the mobilized nutrients through its mycelial network to the roots. Together, these partners synergistically promote the uptake of essential nutrients, including nitrogen (N), phosphorus (P), and potassium (K). (2) The hyphal networks formed by AMF bridge soil pores, thereby enhancing water availability and improving photosynthetic efficiency. (3) Ion homeostasis is maintained through the efflux of sodium ions (Na^+^) and the retention of potassium ions (K^+^), collectively sustaining cellular osmotic balance. (4) Antioxidant enzymes, such as superoxide dismutase (SOD), peroxidase (POD), and catalase (CAT), are crucial in scavenging reactive oxygen species (ROS) to mitigate oxidative damage. (5) The production of antibiotics and lipopeptides, along with the activation of induced systemic resistance (ISR) mechanisms—including polyphenol oxidase (PPO) and lignin biosynthesis—confers biotic resistance against pathogens.

The co-inoculation of AMF and PGPR typically results in synergistic effects that exceed the benefits conferred by single inoculations. This synergy is often mediated by the PGPR-induced enhancement of AMF colonization, which in turn amplifies plant growth and nutrient acquisition (Alam et al., 2011; Wilkes et al., 2020; Savastano and Bais, 2024) (Figure 4). For instance, inoculation with *Bacillus subtilis* was found to significantly increase root colonization by *Funneliformis mosseae* in rose-scented geranium (*Pelargonium graveolens*), leading to increased shoot biomass and improved nutrient uptake compared to those observed in single inoculations or uninoculated controls (Alam et al., 2011). Similarly, another study reported that *Bacillus subtilis* promoted the colonization of onion (*Allium cepa*) by *Rhizophagus irregularis*, which correlated with increased plant biomass and enhanced nutrient acquisition (Toro et al., 1997). Studies on winter wheat have further demonstrated that dual inoculation with *Bacillus amyloliquefaciens* and *Rhizophagus intraradices* increased the number of arbuscules and the production of glomalin—a glycoprotein associated with AMF biomass and soil aggregation—indicating that PGPR exert a stimulatory effect on AMF development (Wilkes et al., 2020). However, the relationship between AMF and PGPR is not universally linear or predictable. A meta-analysis revealed that while dual inoculation consistently improves plant performance, the application of PGPR does not invariably enhance AMF colonization. This finding suggests that alternative mechanisms, including the direct promotion of plant growth by PGPR, modulation of root exudates, or activation of ISR, may also contribute to the observed synergistic effects (Primieri et al., 2022). There are significant knowledge gaps regarding the species-specific interactions between AMF and PGPR, the influence of soil nutrient status on these tripartite associations, and the optimal timing of inoculation for maximizing their benefits (Gopal et al., 2012; Primieri et al., 2022).

### 5.2 Alleviation of abiotic stress through AMF-PGPR cooperation

Abiotic stressors, particularly drought and salinity, significantly restrict plant growth and productivity. The combined application of AMF and PGPR has been shown to be particularly effective in enhancing plant tolerance to these stressors, as their synergistic interactions amplify the beneficial effects beyond those achieved by individual inoculants. PGPR can stimulate the growth and activity of AMF under conditions of water limitation, thereby enhancing the water and nutrient scavenging capacity of the mycorrhizal hyphal network. This synergy results in improved plant physiological status and enhanced drought resilience across various species. For instance, co-inoculation of *Bacillus* sp. with *Funneliformis mosseae* and *Rhizophagus irregularis* was found to mitigate drought stress in lettuce (*Lactuca sativa*) by increasing the colonization of AMF and improving plant water status (Vivas et al., 2003). This co-inoculation also enhanced multiple physiological parameters, including nutrient uptake (particularly nitrogen, phosphorus, and potassium), photosynthetic activity, and Gs, while reducing oxidative damage through the upregulation of antioxidant enzymes (Marulanda et al., 2006; Anli et al., 2020; Begum et al., 2022). The combined application of *Glomus versiforme* and *Bacillus methylotrophicus* not only promoted nutrient uptake in tobacco (*Nicotiana tabacum*), but also increased the activities of antioxidant enzymes, resulting in reduced oxidative stress and increased biomass under drought conditions (Begum et al., 2022). A study on date palms demonstrated that co-inoculation with AMF and PGPR significantly improved nitrogen and phosphorus acquisition, enhanced Gs, and promoted superior morphological traits. These improvements were attributed to the extended AMF hyphal network that facilitated water uptake from the deeper soil layers (Anli et al., 2020). Similarly, the co-inoculation of *Rhizophagus irregularis* and *Bacillus thuringiensis* enhanced water uptake in *Retama sphaerocarpa* through hyphal-mediated water transport (Marulanda et al., 2006). Despite these advancements, the molecular interactions among plants, AMF, and PGPR under drought stress remain largely unexplored (Nanjundappa et al., 2019).

The co-inoculation of AMF and PGPR significantly enhances plant salt tolerance and growth, with tomato serving as a representative model crop in such studies. AMF establish mutualistic associations with plant roots, thereby facilitating the absorption of nutrients, particularly phosphorus, through their extensive hyphal networks. Additionally, AMF improve water retention and soil structure, thereby enhancing plant resilience under saline conditions (Wang et al., 2023; Boyno et al., 2025). In parallel, PGPR support plant growth through phytohormone production, nitrogen fixation, and nutrient solubilization, which collectively promote growth and enhance stress tolerance (Mhlongo et al., 2020). Furthermore, AMF and PGPR interact synergistically to enhance plant osmotic adjustment by promoting the accumulation of proline and soluble sugars. They also jointly activate antioxidant enzyme systems, including SOD, POD, and CAT, to scavenge ROS and mitigate membrane lipid peroxidation (Zhou X. et al., 2022; Bilgili and Bilgili, 2023). AMF and PGPR also co-regulate the expression of ion transport proteins, including high-affinity potassium transporters (HKT) and sodium/hydrogen exchangers (NHX), as well as genes in the salt overly sensitive (SOS) signaling pathway. This coordinated regulation facilitates sodium (Na^+^) efflux and vacuolar compartmentalization, thereby maintaining the intracellular potassium (K^+^)/sodium (Na^+^) balance (Reginato et al., 2021; Chen et al., 2022).

The co-inoculation of PGPR, specifically *Pseudomonas putida* and *Azotobacter chroococcum*, with AMF, such as *Glomus mosseae*, significantly enhances both stem and root dry weights in tomato plants, as well as the uptake of essential nutrients, including phosphorus, magnesium, potassium, and calcium. Notably, their combined application exerts the most pronounced effect on phosphorus content (Zare et al., 2011). Similarly, the co-inoculation of *Funneliformis mosseae* (AMF) and *Bacillus subtilis* (PGPR) alleviates low-salinity stress by strengthening the antioxidant defense system and osmotic adjustments. Here, AMF primarily facilitated the accumulation of osmolytes, whereas PGPR enhanced the total antioxidant capacity, and their synergistic effect increased fruit yield (Zhou X. et al., 2022). Additionally, combined applications of AMF and PGPR, either alone or in conjunction with compost, have been reported to be more effective in alleviating water stress in tomatoes than single biofertilizer treatments. This subsequently increases the activities of antioxidant enzymes in the shoot, improves chlorophyll fluorescence, and enhances the contents of fruit sugar and protein, even under drought conditions (Tahiri et al., 2022). The ecological interactions between AMF and PGPR in the rhizosphere and hyphosphere further underscore their synergistic effects, as the ERM of AMF serves as a conduit for the colonization and dispersal of PGPR, thereby facilitating access to nutrient-rich niches and enhancing the bioavailability of immobilized nutrients, such as organic phosphorus (El-Sawah et al., 2021; Jiang et al., 2021). In turn, PGPR-derived exopolysaccharides improve soil structure, form protective biofilms around roots to mitigate Na^+^ influx, and potentially stimulate the colonization of AMF through signaling interactions (Kong et al., 2020; Qin et al., 2024).

### 5.3 Enhanced biotic stress resistance via AMF-PGPR consortia

The co-inoculation of AMF and plant PGPR enhances plant defense mechanisms against pathogens, producing a synergistic effect that is more effective that of individual inoculants. Both microbial groups can directly antagonize pathogens and/or induce systemic resistance in host plants (Lowe et al., 2012), with AMF competing with soil-borne pathogens for root colonization sites and nutrients. For instance, *Glomus* spp. can decrease infection by the white rot fungus *Sclerotium rolfsii* in common beans through resource competition. Additionally, PGPR such as *Bacillus subtilis* produce antimicrobial compounds, including lipopeptides and antibiotics, which directly inhibit the growth of pathogens, as evidenced in the same pathosystem (Mohamed et al., 2019).

Moreover, co-inoculation with AMF and PGPR can enhance intrinsic defense mechanisms in the host plant. For instance, the co-application of *Glomus* spp. and *Bacillus subtilis* in common beans has been shown to synergistically increase the activity of defense-related enzymes, such as POD and PPO, which are essential for lignin biosynthesis and ROS scavenging (Mohamed et al., 2019). Plant-associated PGPR may also enhance phosphorus availability to plants, thereby supporting both growth and defense during pathogen challenges (Kohler et al., 2007). Notably, although PGPR often promote AMF colonization (Savastano and Bais, 2024), this does not necessarily correlate with improved pathogen resistance. For instance, challenge with *Aspergillus niger* increased the colonization of lettuce by *Rhizophagus irregularis* following co-inoculation with *Bacillus subtilis*; however, this did not result in stronger disease suppression. This suggests the involvement of additional interaction mechanisms, such as the priming of plant immune responses or alterations in the composition of the rhizospheric microbiome (Kohler et al., 2007), necessitating a deeper understanding of microbe–microbe and microbe–plant signaling under pathogen stress (Niu et al., 2020). Although current research often emphasizes the roles of individual microorganisms, the synergistic effects arising from multi-level interactions between AMF and PGPR offer greater potential for enhancing plant stress resistance and productivity. Future efforts should focus on constructing functionally complementary synthetic microbial communities and integrating molecular breeding approaches with the development of targeted microbial agents to provide effective strategies for the green and sustainable development of agriculture, particularly in saline-alkali regions.

## 6 Conclusions and future prospects

The escalating challenges posed by climate change and intensive agricultural practices underscore the need to develop sustainable strategies for maintaining and enhancing tomato productivity. This review provides a comprehensive examination of the multifaceted roles of PGPR and AMF in alleviating both biotic and abiotic stresses in tomato cultivation (Figure 4). These beneficial rhizospheric microorganisms represent a promising biological alternative to conventional chemical inputs, providing the dual benefits of stress mitigation and growth promotion through interconnected physiological, molecular, and ecological mechanisms. PGPR enhance stress tolerance in tomato plants through diverse direct and indirect mechanisms, including biological nitrogen fixation, solubilization of phosphorus and potassium, siderophore-mediated iron acquisition, and the production of phytohormones—particularly auxins, gibberellins, and cytokinins. These bacteria also activate ISR, reinforce cell wall structures by inducing the deposition of lignin and callose, enhance the activities of antioxidant enzymes such as SOD, CAT, and POD, and promote the accumulation of osmoprotectants such as proline and glycine betaine. Concurrently, AMF establish mutualistic symbioses with the roots of tomato plants, extending hyphal networks that enhance the uptake of water and nutrients, particularly phosphorus and nitrogen. AMF also modulate hormonal signaling networks involving ABA, JA, and strigolactones, activate MIR, regulate the expression of *AQP* genes to enhance root hydraulic conductivity, and improve photosynthetic efficiency and WUE under stress conditions.

The synergistic interactions between PGPR and AMF consistently surpass the effects of single inoculations. These microbial partners occupy complementary ecological niches and engage in mutualistic exchanges that enhance their respective functions. PGPR can stimulate spore germination and hyphal development in AMF, whereas AMF hyphae facilitate the colonization and dispersal of PGPR within the soil matrix. This cooperation enhances nutrient acquisition, stress tolerance, and pathogen suppression through multiple mechanisms, including improved antioxidant defense, enhanced osmotic adjustment, and coordinated regulation of ion transporters, including HKT, NHX, and the SOS pathway, as well as the establishment of disease-suppressive rhizospheric microbiomes. Despite substantial progress in understanding plant–microbe interactions, several critical knowledge gaps and challenges remain, warranting further investigation, as discussed hereafter. First, the molecular signaling networks underlying tripartite interactions among tomato plants, PGPR, and AMF under stress conditions remain poorly characterized. Although individual signaling pathways involving phytohormones, ROS, and calcium fluxes have been identified, their integration and cross-talk during combined biotic and abiotic stress episodes require systematic investigation. The integration of advanced omics approaches—including transcriptomics, metabolomics, and proteomics—with spatial analysis techniques could elucidate the temporal and spatial dynamics of these signaling networks.

The translation of laboratory and greenhouse findings to field conditions remains inconsistent, largely due to the complexity of natural soil ecosystems and competition from indigenous microbial communities. Future research should focus on understanding the ecological determinants of inoculant establishment and persistence, including soil organic matter content, pH, nutrient availability, and structure of the resident microbial community. The development of effective formulations and delivery systems that protect microbial viability during storage and following soil application represents a major technological bottleneck, requiring innovative solutions. The integrated application of AMF and PGPR alongside emerging agricultural technologies offers unexplored synergies. The combination of microbial inoculants with precision agriculture tools, including sensor-based monitoring of soil and plant health, could facilitate site-specific and temporally optimized inoculation strategies. Furthermore, the integration of microbiome engineering with genome editing technologies, such as CRISPR-Cas systems, presents opportunities for enhancing beneficial plant–microbe interactions by modifying plant genes involved in symbiotic signaling and nutrient exchange. The development of customized microbial consortia tailored to specific stress scenarios and tomato varieties represents a priority research direction. Unlike single-strain inoculants, synthetic microbial communities can leverage functional complementarity and emergent properties to deliver robust stress protection across diverse environmental conditions. High-throughput screening platforms, coupled with machine learning algorithms, could further accelerate the identification of optimal microbial combinations and their dose–response relationships.

In conclusion, PGPR and AMF represent powerful tools for the sustainable production of tomato under increasing environmental stress. The synergistic potential of these beneficial microorganisms, when effectively harnessed through integrated management strategies, offers a viable strategy for reducing chemical inputs while maintaining or enhancing crop productivity. Achieving this potential will necessitate sustained interdisciplinary collaboration among plant biologists, microbiologists, soil scientists, agronomists, and agricultural engineers to translate mechanistic insights into practical solutions that benefit farmers, consumers, and the environment.

## References

[ref1] Abdel Latef A. A. Chaoxing H. (2011a). Effect of arbuscular mycorrhizal fungi on growth, mineral nutrition, antioxidant enzymes activity and fruit yield of tomato grown under salinity stress. Sci. Hort. 127, 228–233. doi: 10.1016/j.scienta.2010.09.020

[ref2] Abdel Latef A. A. Chaoxing H. (2011b). Arbuscular mycorrhizal influence on growth, photosynthetic pigments, osmotic adjustment and oxidative stress in tomato plants subjected to low temperature stress. Acta Physiol. Plant. 33, 1217–1225. doi: 10.1007/s11738-010-0650-3

[ref3] Adedayo A. A. Babalola O. O. Prigent-Combaret C. Cruz C. Stefan M. Kutu F. . (2022). The application of plant growth-promoting rhizobacteria in *Solanum lycopersicum* production in the agricultural system: a review. PeerJ 10:e13405. doi: 10.7717/peerj.13405, 35669957 PMC9165593

[ref4] Alam M. Khaliq A. Sattar A. Shukla R. S. Anwar M. Dharni S. (2011). Synergistic effect of arbuscular mycorrhizal fungi and *Bacillus subtilis* on the biomass and essential oil yield of rose-scented geranium (*Pelargonium graveolens*). Arch. Agron. Soil Sci. 57, 889–898. doi: 10.1080/03650340.2010.498013

[ref5] Ali B. Hafeez A. Afridi M. S. Javed M. A. Sumaira Suleman F. . (2023). Bacterial-mediated salinity stress tolerance in maize (*Zea mays* L.): a fortunate way toward sustainable agriculture. ACS Omega 8, 20471–20487. doi: 10.1021/acsomega.3c00723 37332827 PMC10275368

[ref6] Al-Karaki G. N. Hammad R. Rusan M. (2001). Response of two tomato cultivars differing in salt tolerance to inoculation with mycorrhizal fungi under salt stress. Mycorrhiza 11, 43–47. doi: 10.1007/s005720100098

[ref7] Anli M. Baslam M. Tahiri A. Raklami A. Symanczik S. Boutasknit A. . (2020). Biofertilizers as strategies to improve photosynthetic apparatus, growth, and drought stress tolerance in the date palm. Front. Plant Sci. 11:516818. doi: 10.3389/fpls.2020.516818, 33193464 PMC7649861

[ref8] Apel K. Hirt H. (2004). Reactive oxygen species: metabolism, oxidative stress, and signal transduction. Annu. Rev. Plant Biol. 55, 373–399. doi: 10.1146/annurev.arplant.55.031903.141701, 15377225

[ref9] Aroca R. Del Mar Alguacil M. Vernieri P. Ruiz-Lozano J. M. (2008). Plant responses to drought stress and exogenous ABA application are modulated differently by mycorrhization in tomato and an ABA-deficient mutant (*sitiens*). Microb. Ecol. 56, 704–719. doi: 10.1007/s00248-008-9390-y, 18443845

[ref10] Augé R. M. (2001). Water relations, drought and vesicular-arbuscular mycorrhizal symbiosis. Mycorrhiza 11, 3–42. doi: 10.1007/s005720100097

[ref11] Augé R. M. Toler H. D. Saxton A. M. (2015). Arbuscular mycorrhizal symbiosis alters stomatal conductance of host plants more under drought than under amply watered conditions: a meta-analysis. Mycorrhiza 25, 13–24. doi: 10.1007/s00572-014-0585-4, 24831020

[ref12] Babikova Z. Gilbert L. Bruce T. J. Birkett M. Caulfield J. C. Woodcock C. . (2013). Underground signals carried through common mycelial networks warn neighbouring plants of aphid attack. Ecol. Lett. 16, 835–843. doi: 10.1111/ele.12115, 23656527

[ref13] Babikova Z. Johnson D. Bruce T. Pickett J. Gilbert L. (2014). Underground allies: how and why do mycelial networks help plants defend themselves? BioEssays 36, 21–26. doi: 10.1002/bies.201300092, 24129903

[ref14] Backer R. Rokem J. S. Ilangumaran G. Lamont J. Praslickova D. Ricci E. . (2018). Plant growth-promoting rhizobacteria: context, mechanisms of action, and roadmap to commercialization of biostimulants for sustainable agriculture. Front. Plant Sci. 9:1473. doi: 10.3389/fpls.2018.01473, 30405652 PMC6206271

[ref15] Bai B. Liu W. Qiu X. Zhang J. Zhang J. Bai Y. (2022). The root microbiome: Community assembly and its contributions to plant fitness. J. Integr. Plant Biol. 64, 230–243. doi: 10.1111/jipb.13226, 35029016

[ref16] Bal A. Chanway C. P. (2012). Evidence of nitrogen fixation in lodgepole pine inoculated with diazotrophic *Paenibacillus polymyxa*. Botany 90, 891–896. doi: 10.1139/B2012-044

[ref17] Balestrini R. Rosso L. C. Veronico P. Melillo M. T. De Luca F. Fanelli E. . (2019). Transcriptomic responses to water deficit and nematode infection in mycorrhizal tomato roots. Front. Microbiol. 10:1807. doi: 10.3389/fmicb.2019.01807, 31456765 PMC6700261

[ref18] Balliu A. Sallaku G. Rewald B. (2015). AMF inoculation enhances growth and improves the nutrient uptake rates of transplanted, salt-stressed tomato seedlings. Sustainability 7, 15967–15981. doi: 10.3390/su71215799

[ref19] Basu A. Prasad P. Das S. N. Kalam S. Sayyed R. Z. Reddy M. S. . (2021). Plant growth promoting rhizobacteria (PGPR) as green bioinoculants: recent developments, constraints, and prospects. Sustainability 13:1140. doi: 10.3390/su13031140

[ref20] Begum N. Wang L. Ahmad H. Akhtar K. Roy R. Khan M. I. . (2022). Co-inoculation of arbuscular mycorrhizal fungi and the plant growth-promoting rhizobacteria improve growth and photosynthesis in tobacco under drought stress by up-regulating antioxidant and mineral nutrition metabolism. Microb. Ecol. 83, 971–988. doi: 10.1007/s00248-021-01815-7 34309697

[ref21] Berendsen R. L. Vismans G. Yu K. Song Y. de Jonge R. Burgman W. P. . (2018). Disease-induced assemblage of a plant-beneficial bacterial consortium. ISME J. 12, 1496–1507. doi: 10.1038/s41396-018-0093-1, 29520025 PMC5956071

[ref22] Berruti A. Borriello R. Della Beffa M. T. Scariot V. Bianciotto V. (2013). Application of nonspecific commercial AMF inocula results in poor mycorrhization in *Camellia japonica* L. Symbiosis 61, 63–76. doi: 10.1007/s13199-013-0258-7

[ref23] Berruti A. Lumini E. Balestrini R. Bianciotto V. (2016). Arbuscular mycorrhizal fungi as natural biofertilizers: let's benefit from past successes. Front. Microbiol. 6:1559. doi: 10.3389/fmicb.2015.01559, 26834714 PMC4717633

[ref24] Bhadrecha P. Bhawana (2023). “Metabolomics and proteomics behind plant growth-promoting potential of rhizobacteria,” in Metabolomics, Proteomes and Gene Editing Approaches in Biofertilizer Industry, eds. Kaur S. Dwibedi V. Sahu P. K. Kocher G. S. (Singapore: Springer), 299–318.

[ref25] Bilgili A. Bilgili A. V. (2023). Comparison of compost, PGPR, and AMF in the biological control of tomato Fusarium wilt disease. Eur. J. Plant Pathol. 167, 771–786. doi: 10.1007/s10658-023-02710-2

[ref26] Bonfante P. Genre A. (2010). Mechanisms underlying beneficial plant–fungus interactions in mycorrhizal symbiosis. Nat. Commun. 1:48. doi: 10.1038/ncomms1046, 20975705

[ref27] Boyno G. Rezaee Danesh Y. Çevik R. Teniz N. Demir S. Demirer Durak E. . (2025). Synergistic benefits of AMF: development of sustainable plant defense system. Front. Microbiol. 16:1551956. doi: 10.3389/fmicb.2025.1551956, 40761278 PMC12319040

[ref28] Brar B. Bala K. Saharan B. S. Sadh P. K. Duhan J. S. (2024). Bio-boosting agriculture: harnessing the potential of fungi-bacteria-plant synergies for crop improvement. Discov. Plants 1:21. doi: 10.1007/s44372-024-00023-0

[ref29] Bright J. P. Maheshwari H. S. Thangappan S. Perveeen K. Bukhari N. A. Mitra D. . (2025). Biofilmed-PGPR: a next-generation bioinoculant for plant growth promotion in rice (*Oryza sativa* L.) under changing climate. Rice Sci. 32, 94–106. doi: 10.1016/j.rsci.2024.08.008

[ref30] Brunetti C. Saleem A. R. Della Rocca G. Emiliani G. De Carlo A. Balestrini R. . (2021). Effects of plant growth-promoting rhizobacteria strains producing ACC deaminase on photosynthesis, isoprene emission, ethylene formation and growth of *Mucuna pruriens* (L.) DC. in response to water deficit. J. Biotechnol. 331, 53–62. doi: 10.1016/j.jbiotec.2021.03.008, 33727083

[ref31] Bucher M. (2007). Functional biology of plant phosphate uptake at root and mycorrhiza interfaces. New Phytol. 173, 11–26. doi: 10.1111/j.1469-8137.2006.01935.x, 17176390

[ref32] Bücking H. Mensah J. A. Fellbaum C. R. (2016). Common mycorrhizal networks and their effect on the bargaining power of the fungal partner in the arbuscular mycorrhizal symbiosis. Commun. Integr. Biol. 9:e1107684. doi: 10.1080/19420889.2015.1107684, 27066184 PMC4802747

[ref33] Cameron D. D. Neal A. L. van Wees S. C. Ton J. (2013). Mycorrhiza-induced resistance: more than the sum of its parts? Trends Plant Sci. 18, 539–545. doi: 10.1016/j.tplants.2013.06.004, 23871659 PMC4194313

[ref34] Cantrell I. C. Linderman R. G. (2001). Preinoculation of lettuce and onion with VA mycorrhizal fungi reduces deleterious effects of soil salinity. Plant Soil 233, 269–281. doi: 10.1023/A:1010564013601

[ref35] Carrión V. J. Cordovez V. Tyc O. Etalo D. W. de Bruijn I. de Jager V. C. L. . (2018). Involvement of Burkholderiaceae and sulfurous volatiles in disease-suppressive soils. ISME J. 12, 2307–2321. doi: 10.1038/s41396-018-0186-x, 29899517 PMC6092406

[ref36] Chandrasekaran M. Boopathi T. Manivannan P. (2021). Comprehensive assessment of ameliorative effects of AMF in alleviating abiotic stress in tomato plants. J. Fungi 7:303. doi: 10.3390/jof7040303, 33921098 PMC8071382

[ref37] Chandrasekaran M. Chanratana M. Kim K. Seshadri S. Sa T. (2019). Impact of arbuscular mycorrhizal fungi on photosynthesis, water status, and gas exchange of plants under salt stress-a meta-analysis. Front. Plant Sci. 10:457. doi: 10.3389/fpls.2019.00457, 31040857 PMC6476944

[ref38] Chang B. Zhao L. Feng Z. Wei F. Zhang Y. Zhang Y. . (2023). Galactosyltransferase GhRFS6 interacting with GhOPR9 involved in defense against *Verticillium wilt* in cotton. Plant Sci. 328:111582. doi: 10.1016/j.plantsci.2022.111582, 36632889

[ref39] Chapelle E. Mendes R. Bakker P. A. Raaijmakers J. M. (2016). Fungal invasion of the rhizosphere microbiome. ISME J. 10, 265–268. doi: 10.1038/ismej.2015.82, 26023875 PMC4681858

[ref40] Chauhan A. Saini R. Sharma J. C. (2021). Plant growth promoting rhizobacteria and their biological properties for soil enrichment and growth promotion. J. Plant Nutr. 45, 273–299. doi: 10.1080/01904167.2021.1952221

[ref41] Chen A. Chen X. Wang H. Liao D. Gu M. Qu H. . (2014). Genome-wide investigation and expression analysis suggest diverse roles and genetic redundancy of Pht1 family genes in response to Pi deficiency in tomato. BMC Plant Biol. 14:61. doi: 10.1186/1471-2229-14-61, 24618087 PMC4007770

[ref44] Cheng X. Wang M. Yuan M. M. Li J. Xiong W. (2023). Editorial: Rhizosphere microbiome engineering for crop cultivation. Front. Bioeng. Biotechnol. 11:1267442. doi: 10.3389/fbioe.2023.1267442, 37727347 PMC10505815

[ref43] Cheng Y. Narayanan M. Shi X. Chen X. Li Z. Ma Y. (2023). Phosphate-solubilizing bacteria: their agroecological function and optimistic application for enhancing agro-productivity. Sci. Total Environ. 901:166468. doi: 10.1016/j.scitotenv.2023.166468, 37619729

[ref42] Chen Q. Deng X. Elzenga J. T. M. van Elsas J. D. (2022). Effect of soil bacteriomes on mycorrhizal colonization by *Rhizophagus irregularis*—interactive effects on maize (*Zea mays* L.) growth under salt stress. Biol. Fertil. Soils 58, 515–525. doi: 10.1007/s00374-022-01636-x

[ref45] Chitarra W. Pagliarani C. Maserti B. Lumini E. Siciliano I. Cascone P. . (2016). Insights on the impact of arbuscular mycorrhizal symbiosis on tomato tolerance to water stress. Plant Physiol. 171, 1009–1023. doi: 10.1104/pp.16.00307, 27208301 PMC4902612

[ref46] Choudhary D. K. Kasotia A. Jain S. Vaishnav A. Kumari S. Sharma K. P. . (2016). Bacterial-mediated tolerance and resistance to plants under abiotic and biotic stresses. J. Plant Growth Regul. 35, 276–300. doi: 10.1007/s00344-015-9521-x

[ref47] Comby M. Mustafa G. Magnin-Robert M. Randoux B. Fontaine J. Reignault P. . (2017). “Arbuscular mycorrhizal fungi as potential bioprotectants against aerial phytopathogens and pests,” in Arbuscular Mycorrhizas and Stress Tolerance of Plants, ed. Wu Q.-S. (Singapore: Springer), 195–223.

[ref48] Dasgan H. Y. Aktas H. Abak K. Cakmak I. (2002). Determination of screening techniques to salinity tolerance in tomatoes and investigation of genotype responses. Plant Sci. 163, 695–703. doi: 10.1016/S0168-9452(02)00091-2

[ref49] Davies F. T. Jr. Svenson S. E. Cole J. C. Phavaphutanon L. Duray S. A. Olalde-Portugal V. . (1996). Non-nutritional stress acclimation of mycorrhizal woody plants exposed to drought. Tree Physiol. 16, 985–993. doi: 10.1093/treephys/16.11-12.985 14871792

[ref50] de Andrade L. A. Santos C. H. B. Frezarin E. T. Sales L. R. Rigobelo E. C. (2023). Plant growth-promoting rhizobacteria for sustainable agricultural production. Microorganisms 11:1088. doi: 10.3390/microorganisms11041088, 37110511 PMC10146397

[ref53] Degon Z. Dixon S. Rahmatallah Y. Galloway M. Gulutzo S. Price H. . (2023). *Azospirillum brasilense* improves rice growth under salt stress by regulating the expression of key genes involved in salt stress response, abscisic acid signaling, and nutrient transport, among others. Front. Agron. 5:1216503. doi: 10.3389/fagro.2023.1216503, 38223701 PMC10785826

[ref54] Delaeter M. Magnin-Robert M. Randoux B. Lounès-Hadj Sahraoui A. (2024). Arbuscular mycorrhizal fungi as biostimulant and biocontrol agents: a review. Microorganisms 12:1281. doi: 10.3390/microorganisms12071281, 39065050 PMC11278648

[ref55] Dell'Amico J. Torrecillas A. Rodríguez P. Morte A. Sánchez-Blanco M. J. (2002). Responses of tomato plants associated with the arbuscular mycorrhizal fungus *Glomus clarum* during drought and recovery. J. Agric. Sci. 138, 387–393. doi: 10.1017/S0021859602002101

[ref51] De Román M. Fernández I. Wyatt T. Sahrawy M. Heil M. Pozo M. J. (2011). Elicitation of foliar resistance mechanisms transiently impairs root association with arbuscular mycorrhizal fungi. J. Ecol. 99, 36–45. doi: 10.1111/j.1365-2745.2010.01752.x

[ref52] de Vries F. T. Griffiths R. I. Knight C. G. Nicolitch O. Williams A. (2020). Harnessing rhizosphere microbiomes for drought-resilient crop production. Science 368, 270–274. doi: 10.1126/science.aaz5192, 32299947

[ref56] Dhillon J. S. Eickhoff E. M. Mullen R. W. Raun W. R. (2019). World potassium use efficiency in cereal crops. Agron. J. 111, 889–896. doi: 10.2134/agronj2018.07.0462

[ref57] Dimkpa C. Weinand T. Asch F. (2009). Plant–rhizobacteria interactions alleviate abiotic stress conditions. Plant Cell Environ. 32, 1682–1694. doi: 10.1111/j.1365-3040.2009.02028.x, 19671096

[ref58] dos Santos R. M. Diaz P. A. E. Lobo L. L. B. Rigobelo E. C. (2020). Use of plant growth-promoting rhizobacteria in maize and sugarcane: characteristics and applications. Front. Sustain. Food Syst. 4:136. doi: 10.3389/fsufs.2020.00136

[ref60] Duan S. Feng G. Limpens E. Bonfante P. Xie X. Zhang L. (2024). Cross-kingdom nutrient exchange in the plant–arbuscular mycorrhizal fungus–bacterium continuum. Nat. Rev. Microbiol. 22, 773–790. doi: 10.1038/s41579-024-01073-7, 39014094

[ref61] Duc N. H. Csintalan Z. Posta K. (2018). Arbuscular mycorrhizal fungi mitigate negative effects of combined drought and heat stress on tomato plants. Plant Physiol. Biochem. 132, 297–307. doi: 10.1016/j.plaphy.2018.09.011, 30245343

[ref59] Du N. Guo H. Fu R. Dong X. Xue D. Piao F. (2022). Comparative transcriptome analysis and genetic methods revealed the biocontrol mechanism of *Paenibacillus polymyxa* NSY50 against tomato Fusarium wilt. Int. J. Mol. Sci. 23:10907. doi: 10.3390/ijms231810907, 36142825 PMC9501285

[ref62] Ebrahim M. K. Saleem A.-R. (2017). Alleviating salt stress in tomato inoculated with mycorrhizae: photosynthetic performance and enzymatic antioxidants. J. Taibah Univ. Sci. 11, 850–860. doi: 10.1016/j.jtusci.2017.02.002

[ref64] El-Saadony M. T. Saad A. M. Soliman S. M. Salem H. M. Ahmed A. I. Mahmood M. . (2022). Plant growth-promoting microorganisms as biocontrol agents of plant diseases: mechanisms, challenges and future perspectives. Front. Plant Sci. 13:923880. doi: 10.3389/fpls.2022.923880, 36275556 PMC9583655

[ref63] EL Sabagh A. Islam M. S. Hossain A. Iqbal M. A. Mubeen M. Waleed M. . (2022). Phytohormones as growth regulators during abiotic stress tolerance in plants. Front. Agron. 4:765068. doi: 10.3389/fagro.2022.765068

[ref65] El-Sawah A. M. Ali E. K. Ali D. F. Ibrahim H. M. Ei-Sheikh M. A. Sheteiwy M. S. (2021). Arbuscular mycorrhizal fungi and plant growth promoting rhizobacteria enhance soil key enzymes, plant growth, seed yield, and qualitative attributes of guar. Agriculture 11:194. doi: 10.3390/agriculture11030194

[ref66] Evelin H. Kapoor R. Giri B. (2009). Arbuscular mycorrhizal fungi in alleviation of salt stress: a review. Ann. Bot. 104, 1263–1280. doi: 10.1093/aob/mcp251, 19815570 PMC2778396

[ref67] Farhaoui A. Taoussi M. Laasli S. E. Legrifi I. El Mazouni N. Meddich A. . (2025). Arbuscular mycorrhizal fungi and their role in plant disease control: a state-of-the-art. The Microbe 8:100438. doi: 10.1016/j.microb.2025.100438

[ref68] Fasusi O. A. Cruz C. Babalola O. O. (2021). Agricultural sustainability: microbial biofertilizers in rhizosphere management. Agriculture 11:163. doi: 10.3390/agriculture11020163

[ref69] Ferreira C. M. H. Vilas-Boas Â. Sousa C. A. Soares H. M. V. M. Soares E. V. (2019). Comparison of five bacterial strains producing siderophores with ability to chelate iron under alkaline conditions. AMB Express 9:78. doi: 10.1186/s13568-019-0796-3, 31139942 PMC6538730

[ref70] Fester T. Hause G. (2005). Accumulation of reactive oxygen species in arbuscular mycorrhizal roots. Mycorrhiza 15, 373–379. doi: 10.1007/s00572-005-0363-4, 15875223

[ref71] Garcia-Lemos A. M. Großkinsky D. K. Saleem Akhtar S. Nicolaisen M. H. Roitsch T. Nybroe O. . (2020). Identification of root-associated bacteria that influence plant physiology, increase seed germination, or promote growth of the Christmas tree species Abies nordmanniana. Front. Microbiol. 11:566613. doi: 10.3389/fmicb.2020.566613, 33281762 PMC7705201

[ref72] Ge J. Li D. Ding J. Xiao X. Liang Y. (2023). Microbial coexistence in the rhizosphere and the promotion of plant stress resistance: A review. Environ. Res. 222:115298. doi: 10.1016/j.envres.2023.115298, 36642122

[ref73] Genre A. Lanfranco L. Perotto S. Bonfante P. (2020). Unique and common traits in mycorrhizal symbioses. Nat. Rev. Microbiol. 18, 649–660. doi: 10.1038/s41579-020-0402-3, 32694620

[ref74] Glick B. R. (2012). Plant growth-promoting bacteria: mechanisms and applications. Scientifica 2012:963401. doi: 10.6064/2012/963401, 24278762 PMC3820493

[ref75] Golldack D. Li C. Mohan H. Probst N. (2014). Tolerance to drought and salt stress in plants: Unraveling the signaling networks. Front. Plant Sci. 5:151. doi: 10.3389/fpls.2014.00151, 24795738 PMC4001066

[ref76] Gómez-Godínez L. J. Aguirre-Noyola J. L. Martínez-Romero E. Arteaga-Garibay R. I. Ireta-Moreno J. Ruvalcaba-Gómez J. M. (2023). A look at plant-growth-promoting bacteria. Plants 12:1668. doi: 10.3390/plants12081668, 37111891 PMC10145503

[ref78] Gong T. Xin X. F. (2021). Phyllosphere microbiota: Community dynamics and its interaction with plant hosts. J. Integr. Plant Biol. 63, 297–304. doi: 10.1111/jipb.13060, 33369158

[ref77] Gong Y. Chen L. J. Pan S. Y. Li X. W. Xu M. J. Zhang C. M. . (2020). Antifungal potential evaluation and alleviation of salt stress in tomato seedlings by a halotolerant plant growth-promoting actinomycete *Streptomyces* sp. KLBMP5084. Rhizosphere 16:100262. doi: 10.1016/j.rhisph.2020.100262

[ref79] Gopal S. Chandrasekaran M. Shagol C. Kim K.-Y. Sa T.-M. (2012). Spore associated bacteria (SAB) of arbuscular mycorrhizal fungi (AMF) and plant growth promoting rhizobacteria (PGPR) increase nutrient uptake and plant growth under stress conditions. Korean J. Soil Sci. Fertil. 45, 582–592. doi: 10.7745/KJSSF.2012.45.4.582

[ref80] Govindasamy P. Muthusamy S. K. Bagavathiannan M. Mowrer J. Jagannadham P. T. K. Maity A. . (2023). Nitrogen use efficiency-a key to enhance crop productivity under a changing climate. Front. Plant Sci. 14:1121073. doi: 10.3389/fpls.2023.1121073, 37143873 PMC10151540

[ref81] Großkinsky D. K. Tafner R. Moreno M. V. Stenglein S. A. de García Salamone I. E. Nelson L. M. . (2016). Cytokinin production by *Pseudomonas fluorescens* G20-18 determines biocontrol activity against *Pseudomonas syringae* in Arabidopsis. Sci. Rep. 6:23310. doi: 10.1038/srep23310, 26984671 PMC4794740

[ref82] Grover M. Bodhankar S. Sharma A. Sharma P. Singh J. Nain L. (2021). PGPR mediated alterations in root traits: way toward sustainable crop production. Front. Sustain. Food Syst. 4:618230. doi: 10.3389/fsufs.2020.618230

[ref83] Gupta P. Kumar V. Usmani Z. Rani R. Chandra A. Gupta V. K. (2020). Implications of plant growth promoting Klebsiella sp. CPSB4 and Enterobacter sp. CPSB49 in luxuriant growth of tomato plants under chromium stress. Chemosphere 240:124944. doi: 10.1016/j.chemosphere.2019.124944, 31726591

[ref84] Haghpanah M. Hashemipetroudi S. Arzani A. Araniti F. (2024). Drought tolerance in plants: physiological and molecular responses. Plants 13:2962. doi: 10.3390/plants13212962, 39519881 PMC11548289

[ref85] Hajiboland R. Aliasgharzadeh N. Laiegh S. F. Poschenrieder C. (2010). Colonization with arbuscular mycorrhizal fungi improves salinity tolerance of tomato (*Solanum lycopersicum* L.) plants. Plant Soil 331, 313–327. doi: 10.1007/s11104-009-0255-z

[ref86] Hakim S. Naqqash T. Nawaz M. S. Laraib I. Siddique M. J. Zia R. . (2021). Rhizosphere engineering with plant growth-promoting microorganisms for agriculture and ecological sustainability. Front. Sustain. Food Syst. 5:617157. doi: 10.3389/fsufs.2021.617157

[ref89] Handa Y. Nishide H. Takeda N. Suzuki Y. Kawaguchi M. Saito K. (2015). RNA-seq transcriptional profiling of an arbuscular mycorrhiza provides insights into regulated and coordinated gene expression in *Lotus japonicus* and *Rhizophagus irregularis*. Plant Cell Physiol. 56, 1490–1511. doi: 10.1093/pcp/pcv071, 26009592

[ref88] Han Q. Tan W. Zhao Y. Yang F. Yao X. Lin H. . (2022). Salicylic acid-activated BIN2 phosphorylation of TGA3 promotes Arabidopsis PR gene expression and disease resistance. EMBO J. 41:e110682. doi: 10.15252/embj.2022110682, 35950443 PMC9531300

[ref87] Han Y. Lou X. Zhang W. Xu T. Tang M. (2022). Arbuscular mycorrhizal fungi enhanced drought resistance of Populus cathayana by regulating the 14-3-3 family protein genes. Microbiol. Spectrum 10:e0245621. doi: 10.1128/spectrum.02456-21, 35612316 PMC9241863

[ref90] Harrier L. A. Watson C. A. (2004). The potential role of arbuscular mycorrhizal (AM) fungi in the bioprotection of plants against soil-borne pathogens in organic and/or other sustainable farming systems. Pest Manag. Sci. 60, 149–157. doi: 10.1002/ps.820, 14971681

[ref91] Hasanuzzaman M. Bhuyan M. H. M. B. Zulfiqar F. Raza A. Mohsin S. M. Mahmud J. A. . (2020). Reactive oxygen species and antioxidant defense in plants under abiotic stress: revisiting the crucial role of a universal defense regulator. Antioxidants 9:681. doi: 10.3390/antiox9080681, 32751256 PMC7465626

[ref92] Hasanuzzaman M. Parvin K. Bardhan K. Nahar K. Anee T. I. Masud A. A. C. . (2021). Biostimulants for the regulation of reactive oxygen species metabolism in plants under abiotic stress. Cells 10:2537. doi: 10.3390/cells10102537, 34685517 PMC8533957

[ref93] Hassen A. I. Babalola O. O. Carlson R. (2023). “Rhizobacterial-mediated interactions for enhanced symbiotic performance of the root nodule rhizobia in legumes,” in Sustainable Agrobiology, eds. Maheshwari D. K. Dheeman S. (Singapore: Springer), 53–79.

[ref95] Heidel A. J. Baldwin I. T. (2004). Microarray analysis of salicylic acid- and jasmonic acid-signalling in responses of *Nicotiana attenuata* to attack by insects from multiple feeding guilds. Plant Cell Environ. 27, 1362–1373. doi: 10.1111/j.1365-3040.2004.01228.x

[ref96] Heo A. Y. Koo Y. M. Choi H. W. (2022). Biological control activity of plant growth promoting rhizobacteria *Burkholderia contaminans* AY001 against tomato *Fusarium wilt* and bacterial speck diseases. Biology 11:619. doi: 10.3390/biology11040619, 35453817 PMC9028202

[ref97] Herrera-Medina M. J. Steinkellner S. Vierheilig H. Ocampo Bote J. A. García Garrido J. M. (2007). Abscisic acid determines arbuscule development and functionality in the tomato arbuscular mycorrhiza. New Phytol. 175, 554–564. doi: 10.1111/j.1469-8137.2007.02107.x, 17635230

[ref94] He Z. He C. Zhang Z. Zou Z. Wang H. (2007). Changes of antioxidative enzymes and cell membrane osmosis in tomato colonized by arbuscular mycorrhizae under NaCl stress. Colloids Surf. B Biointerfaces 59, 128–133. doi: 10.1016/j.colsurfb.2007.04.023, 17560092

[ref98] Hildebrandt U. Regvar M. Bothe H. (2007). Arbuscular mycorrhiza and heavy metal tolerance. Phytochemistry 68, 139–146. doi: 10.1016/j.phytochem.2006.09.023 17078985

[ref99] Jaiti F. Meddich A. El Hadrami I. (2008). Effectiveness of arbuscular mycorrhizal fungi in the protection of date palm (*Phoenix dactylifera* L.) against bayoud disease. Physiol. Mol. Plant Pathol. 71, 166–173. doi: 10.1016/j.pmpp.2008.01.002

[ref100] Jambon I. Thijs S. Weyens N. Vangronsveld J. (2018). Harnessing plant-bacteria-fungi interactions to improve plant growth and degradation of organic pollutants. J. Plant Interact. 13, 119–130. doi: 10.1080/17429145.2018.1441450

[ref101] Javot H. Penmetsa R. V. Terzaghi N. Cook D. R. Harrison M. J. (2007). A *Medicago truncatula* phosphate transporter indispensable for the arbuscular mycorrhizal symbiosis. Proc. Natl. Acad. Sci. USA 104, 1720–1725. doi: 10.1073/pnas.0608136104, 17242358 PMC1785290

[ref102] Jha Y. Subramanian R. B. (2014). PGPR regulate caspase-like activity, programmed cell death, and antioxidant enzyme activity in paddy under salinity. Physiol. Mol. Biol. Plants 20, 201–207. doi: 10.1007/s12298-014-0224-8, 24757324 PMC3988331

[ref103] Jiang F. Zhang L. Zhou J. George T. S. Feng G. (2021). Arbuscular mycorrhizal fungi enhance mineralisation of organic phosphorus by carrying bacteria along their extraradical hyphae. New Phytol. 230, 304–315. doi: 10.1111/nph.17081, 33205416

[ref104] Johan P. D. Ahmed O. H. Omar L. Hasbullah N. A. (2021). Phosphorus transformation in soils following co-application of charcoal and wood ash. Agronomy 11:2010. doi: 10.3390/agronomy11102010

[ref105] Johnson D. Gilbert L. (2015). Interplant signalling through hyphal networks. New Phytol. 205, 1448–1453. doi: 10.1111/nph.13115, 25421970

[ref106] Jomova K. Alomar S. Y. Alwasel S. H. Nepovimova E. Kuca K. Valko M. (2024). Several lines of antioxidant defense against oxidative stress: antioxidant enzymes, nanomaterials with multiple enzyme-mimicking activities, and low-molecular-weight antioxidants. Arch. Toxicol. 98, 1323–1367. doi: 10.1007/s00204-024-03696-4, 38483584 PMC11303474

[ref107] Joshi H. Bisht N. Mishra S. K. Prasad V. Chauhan P. S. (2023). *Bacillus amyloliquefaciens* modulate carbohydrate metabolism in rice-PGPR cross-talk under abiotic stress and phytohormone treatments. J. Plant Growth Regul. 42, 4466–4483. doi: 10.1007/s00344-023-10913-4

[ref108] Jung S. C. Martinez-Medina A. Lopez-Raez J. A. Pozo M. J. (2012). Mycorrhiza-induced resistance and priming of plant defenses. J. Chem. Ecol. 38, 651–664. doi: 10.1007/s10886-012-0134-6, 22623151

[ref109] Kalayu G. (2019). Phosphate solubilizing microorganisms: promising approach as biofertilizers. Int. J. Agron. 2019:4917256. doi: 10.1155/2019/4917256

[ref110] Kaundal S. Rana N. Kumar Y. Alhewairini S. S. Barasarathi J. Haron F. F. . (2025). Biofilmed multifarious rhizobacterial isolates of tomato rhizosphere of North-Western Himalayas promote plant growth in tomato. Front. Plant Sci. 16:1610707. doi: 10.3389/fpls.2025.1610707, 40666299 PMC12259571

[ref111] Kaur S. Samota M. K. Choudhary M. Choudhary M. Pandey A. K. Sharma A. . (2022). How do plants defend themselves against pathogens-Biochemical mechanisms and genetic interventions. Physiol. *Mol. Biol. Plants* 28, 485–504. doi: 10.1007/s12298-022-01146-y, 35400890 PMC8943088

[ref112] Ketut Widnyana I. (2019). “PGPR (Plant Growth Promoting Rizobacteria) benefits in spurring germination, growth and increase the yield of tomato plants,” in Recent Advances in Tomato Breeding and Production, eds. Nyaku S. T. Danquah A. (London: IntechOpen).

[ref114] Khan F. Siddique A. B. Shabala S. Zhou M. Zhao C. (2023). Phosphorus plays key roles in regulating plants' physiological responses to abiotic stresses. Plants 12:2861. doi: 10.3390/plants12152861, 37571014 PMC10421280

[ref113] Khan N. Ali S. Shahid M. A. Mustafa A. Sayyed R. Z. Curá J. A. (2021). Insights into the interactions among roots, rhizosphere, and rhizobacteria for improving plant growth and tolerance to abiotic stresses: a review. Cells 10:1551. doi: 10.3390/cells10061551, 34205352 PMC8234610

[ref115] Kim B. Park A. R. Song C. W. Song H. Kim J. C. (2022). Biological control efficacy and action mechanism of *Klebsiella pneumoniae* JCK-2201 producing meso-2,3-butanediol against tomato bacterial wilt. Front. Microbiol. 13:914589. doi: 10.3389/fmicb.2022.914589, 35910601 PMC9333516

[ref116] Klimasmith I. M. Kent A. D. (2022). Micromanaging the nitrogen cycle in agroecosystems. Trends Microbiol. 30, 1045–1055. doi: 10.1016/j.tim.2022.04.006, 35618540

[ref117] Kohler J. Caravaca F. Carrasco L. Roldán A. (2007). Interactions between a plant growth-promoting rhizobacterium, an AM fungus and a phosphate-solubilising fungus in the rhizosphere of *Lactuca sativa*. Appl. Soil Ecol. 35, 480–487. doi: 10.1016/j.apsoil.2006.10.006

[ref118] Kong L. Gong X. Zhang X. Zhang W. Sun J. Chen B. (2020). Effects of arbuscular mycorrhizal fungi on photosynthesis, ion balance of tomato plants under saline-alkali soil condition. J. Plant Nutr. 43, 682–698. doi: 10.1080/01904167.2019.1701029

[ref119] Koprivova A. Schuck S. Jacoby R. P. Klinkhammer I. Welter B. Leson L. . (2019). Root-specific camalexin biosynthesis controls the plant growth-promoting effects of multiple bacterial strains. Proc. Natl. Acad. Sci. USA 116, 15735–15744. doi: 10.1073/pnas.1818604116, 31311863 PMC6681745

[ref120] Kousar B. Bano A. Khan N. (2020). PGPR modulation of secondary metabolites in tomato infested with *Spodoptera litura*. Agronomy 10:778. doi: 10.3390/agronomy10060778

[ref121] Kudoyarova G. Arkhipova T. Korshunova T. Bakaeva M. Loginov O. Dodd I. C. (2019). Phytohormone mediation of interactions between plants and non-symbiotic growth promoting bacteria under edaphic stresses. Front. Plant Sci. 10:1368. doi: 10.3389/fpls.2019.01368, 31737004 PMC6828943

[ref125] Kumari B. Mallick M. A. Solanki M. K. Solanki A. C. Hora A. Guo W. (2019). “Plant growth promoting rhizobacteria (PGPR): modern prospects for sustainable agriculture,” in Plant Health Under Biotic Stress, eds. Ansari R. Mahmood I. (Singapore: Springer), 1–20.

[ref126] Kumari S. M. P. Prabina B. J. (2019). Protection of tomato, *Lycopersicon esculentum* from wilt pathogen, *Fusarium oxysporum* f.sp. *lycopersici* by arbuscular mycorrhizal fungi, *Glomus* sp. Int. J. Curr. Microbiol. Appl. Sci. 8, 1368–1378. doi: 10.20546/ijcmas.2019.804.159

[ref124] Kumar M. Tomar M. Bhuyan D. J. Punia S. Grasso S. Sá A. G. A. . (2021). Tomato (*Solanum lycopersicum* L.) seed: a review on bioactives and biomedical activities. Biomed. Pharmacother. 142:112018. doi: 10.1016/j.biopha.2021.112018, 34449317

[ref123] Kumar R. Sagar V. Verma V. C. Kumari M. Gujjar R. S. Goswami S. K. . (2023). Drought and salinity stresses induced physio-biochemical changes in sugarcane: an overview of tolerance mechanism and mitigating approaches. Front. Plant Sci. 14:1225234. doi: 10.3389/fpls.2023.1225234, 37645467 PMC10461627

[ref122] Kumar V. Nautiyal C. S. (2022). Plant abiotic and biotic stress alleviation: from an endophytic microbial perspective. Curr. Microbiol. 79:311. doi: 10.1007/s00284-022-03012-2, 36088388

[ref127] Lanza M. G. D. B. Reis A. R. D. (2021). Roles of selenium in mineral plant nutrition: ROS scavenging responses against abiotic stresses. Plant Physiol. Biochem. 164, 27–43. doi: 10.1016/j.plaphy.2021.04.026, 33962229

[ref128] Lax P. Becerra A. G. Soteras F. Cabello M. Doucet M. E. (2011). Effect of the arbuscular mycorrhizal fungus *Glomus intraradices* on the false root-knot nematode *Nacobbus aberrans* in tomato plants. Biol. Fertil. Soils 47, 591–597. doi: 10.1007/s00374-010-0514-4

[ref129] Lenoir I. Fontaine J. Lounès-Hadj Sahraoui A. (2016). Arbuscular mycorrhizal fungal responses to abiotic stresses: a review. Phytochemistry 123, 4–15. doi: 10.1016/j.phytochem.2016.01.002 26803396

[ref133] Lian T. Ma Q. Shi Q. Cai Z. Zhang Y. Cheng Y. . (2019). High aluminum stress drives different rhizosphere soil enzyme activities and bacterial community structure between aluminum-tolerant and aluminum-sensitive soybean genotypes. Plant Soil 440, 409–425. doi: 10.1007/s11104-019-04089-8

[ref130] Li M. Hou S. Wang J. Hu J. Lin X. (2021). Arbuscular mycorrhizal fungus suppresses tomato (*Solanum lycopersicum* Mill) *Ralstonia* wilt via establishing a soil-plant integrated defense system. J. Soils Sediments 21, 3607–3619. doi: 10.1007/s11368-021-03016-8

[ref134] Lin J. Wang Y. Sun S. Mu C. Yan X. (2017). Effects of arbuscular mycorrhizal fungi on the growth, photosynthesis and photosynthetic pigments of Leymus chinensis seedlings under salt-alkali stress and nitrogen deposition. Sci. Total Environ. 576, 234–241. doi: 10.1016/j.scitotenv.2016.10.091, 27788438

[ref132] Li Q. Shen H. Yuan S. Dai X. Yang C. (2023). miRNAs and lncRNAs in tomato: Roles in biotic and abiotic stress responses. Front. Plant Sci. 13:1094459. doi: 10.3389/fpls.2022.1094459, 36714724 PMC9875070

[ref136] Liu D. Shen Z. Zhuang K. Qiu Z. Deng H. Ke Q. . (2022). Systematic annotation reveals CEP function in tomato root development and abiotic stress response. Cells 11:2935. doi: 10.3390/cells11192935, 36230896 PMC9562649

[ref135] Liu J. Maldonado-Mendoza I. Lopez-Meyer M. Cheung F. Town C. D. Harrison M. J. (2007). Arbuscular mycorrhizal symbiosis is accompanied by local and systemic alterations in gene expression and an increase in disease resistance in the shoots. Plant J. 50, 529–544. doi: 10.1111/j.1365-313X.2007.03069.x, 17419842

[ref131] Li W. Li W. B. Xing L. J. Guo S. X. (2023). Effect of arbuscular mycorrhizal fungi (AMF) and plant growth-promoting rhizobacteria (PGPR) on microorganism of phenanthrene and pyrene contaminated soils. Int. J. Phytoremediation 25, 240–251. doi: 10.1080/15226514.2022.2071832 35549569

[ref137] Lopes M. J. D. S. Dias-Filho M. B. Gurgel E. S. C. (2021). Successful plant growth-promoting microbes: inoculation methods and abiotic factors. Front. Sustain. Food Syst. 5:606454. doi: 10.3389/fsufs.2021.606454

[ref138] Lowe A. Rafferty-McArdle S. M. Cassells A. C. (2012). Effects of AMF- and PGPR-root inoculation and a foliar chitosan spray in single and combined treatments on powdery mildew disease in strawberry. Agric. Food Sci. 21, 28–38. doi: 10.23986/afsci.4997

[ref139] Madhaiyan M. Poonguzhali S. Sa T. (2007). Metal tolerating methylotrophic bacteria reduces nickel and cadmium toxicity and promotes plant growth of tomato (*Lycopersicon esculentum* L). Chemosphere 69, 220–228. doi: 10.1016/j.chemosphere.2007.04.017, 17512031

[ref140] Mannino G. Nerva L. Gritli T. Novero M. Fiorilli V. Bacem M. . (2020). Effects of different microbial inocula on tomato tolerance to water deficit. Agronomy 10:170. doi: 10.3390/agronomy10020170

[ref141] Martínez-Viveros O. Jorquera M. A. Crowley D. E. Gajardo G. Mora M. L. (2010). Mechanisms and practical considerations involved in plant growth promotion by rhizobacteria. J. Soil Sci. Plant Nutr. 10, 293–319. doi: 10.4067/S0718-95162010000100006

[ref142] Martín-Rodríguez J. A. León-Morcillo R. Vierheilig H. Ocampo Bote J. A. Ludwig-Müllerm J. García-Garrido J. M. . (2010). Mycorrhization of the notabilis and sitiens tomato mutants in relation to abscisic acid and ethylene contents. J. Plant Physiol. 167, 606–613. doi: 10.1016/j.jplph.2009.11.014 20079554

[ref143] Marulanda A. Barea J. M. Azcón R. (2006). An indigenous drought-tolerant strain of *Glomus intraradices* associated with a native bacterium improves water transport and root development in *Retama sphaerocarpa*. Microb. Ecol. 52, 670–678. doi: 10.1007/s00248-006-9078-0, 17075734

[ref144] Mazumder S. Bhattacharya D. Lahiri D. Nag M. (2025). Rhizobacteria and arbuscular mycorrhizal fungi (AMF) community in growth management and mitigating stress in millets: A plant-soil microbe symbiotic relationship. Curr. Microbiol. 82:242. doi: 10.1007/s00284-025-04230-0, 40220175

[ref145] Meena M. Swapnil P. Divyanshu K. Kumar S. Harish Tripathi Y. N. . (2020). PGPR-mediated induction of systemic resistance and physiochemical alterations in plants against the pathogens: current perspectives. J. Basic Microbiol. 60, 828–861. doi: 10.1002/jobm.202000370 32815221

[ref146] Mekkaoui F. Ait-El-Mokhtar M. Zaari Jabri N. Amghar I. Essadssi S. Hmyene A. (2024). The use of compost and arbuscular mycorrhizal fungi and their combination to improve tomato tolerance to salt stress. Plants 13:2225. doi: 10.3390/plants13162225, 39204661 PMC11359464

[ref147] Mekonnen H. Kibret M. (2021). The roles of plant growth promoting rhizobacteria in sustainable vegetable production in Ethiopia. Chem. Biol. Technol. Agric. 8:15. doi: 10.1186/s40538-021-00213-y

[ref148] Melini F. Melini V. Luziatelli F. Abou Jaoudé R. Ficca A. G. Ruzzi M. (2023). Effect of microbial plant biostimulants on fruit and vegetable quality: current research lines and future perspectives. Front. Plant Sci. 14:1251544. doi: 10.3389/fpls.2023.1251544, 37900743 PMC10602749

[ref149] Mendes R. Kruijt M. de Bruijn I. Dekkers E. van der Voort M. Schneider J. H. . (2011). Deciphering the rhizosphere microbiome for disease-suppressive bacteria. Science 332, 1097–1100. doi: 10.1126/science.1203980, 21551032

[ref150] Mhamdi A. (2023). Hydrogen peroxide in plants. Adv. Bot. Res. 105, 43–75. doi: 10.1016/bs.abr.2022.11.002

[ref151] Mhlongo M. I. Piater L. A. Dubery I. A. (2022). Profiling of volatile organic compounds from four plant growth-promoting rhizobacteria by SPME-GC–MS: A metabolomics study. Meta 12:763. doi: 10.3390/metabo12080763, 36005635 PMC9414699

[ref152] Mhlongo M. I. Piater L. A. Steenkamp P. A. Labuschagne N. Dubery I. A. (2020). Metabolic profiling of PGPR-treated tomato plants reveal priming-related adaptations of secondary metabolites and aromatic amino acids. Meta 10:210. doi: 10.3390/metabo10050210, 32443694 PMC7281251

[ref9001] Minchev Z. Kostenko O. Soler R. Pozo M. J. (2021). Microbial consortia for effective biocontrol of root and foliar diseases in tomato. Front. Plant Sci. 12, 756368. doi: 10.3389/fpls.2021.756368 34804094 PMC8602810

[ref153] Mohamed I. Eid K. E. Abbas M. H. H. Salem A. A. Ahmed N. Ali M. . (2019). Use of plant growth promoting Rhizobacteria (PGPR) and mycorrhizae to improve the growth and nutrient utilization of common bean in a soil infected with white rot fungi. Ecotoxicol. Environ. Saf. 171, 539–548. doi: 10.1016/j.ecoenv.2018.12.100, 30641315

[ref154] Mohanty P. Singh P. K. Chakraborty D. Mishra S. Pattnaik R. (2021). Insight into the role of PGPR in sustainable agriculture and environment. Front. Sustain. Food Syst. 5:667150. doi: 10.3389/fsufs.2021.667150

[ref155] Mokrani S. Nabti E.-h. Cruz C. (2020). Current advances in plant growth promoting bacteria alleviating salt stress for sustainable agriculture. Appl. Sci. 10:7025. doi: 10.3390/app10207025

[ref156] Molnár Z. Solomon W. Mutum L. Janda T. (2023). Understanding the mechanisms of Fe deficiency in the rhizosphere to promote plant resilience. Plants 12:1945. doi: 10.3390/plants12101945, 37653862 PMC10224236

[ref157] Muhammad Aslam M. Waseem M. Jakada B. H. Okal E. J. Lei Z. Saqib H. S. A. . (2022). Mechanisms of abscisic acid-mediated drought stress responses in plants. Int. J. Mol. Sci. 23:1084. doi: 10.3390/ijms23031084, 35163008 PMC8835272

[ref158] Mukherjee A. Gaurav A. K. Singh S. Yadav S. Bhowmick S. Abeysinghe S. . (2022). The bioactive potential of phytohormones: a review. Biotechnol. Rep. 35:e00748. doi: 10.1016/j.btre.2022.e00748, 35719852 PMC9204661

[ref159] Nacoon S. Jogloy S. Riddech N. Mongkolthanaruk W. Kuyper T. W. Boonlue S. (2020). Interaction between phosphate solubilizing bacteria and arbuscular mycorrhizal fungi on growth promotion and tuber inulin content of *Helianthus tuberosus* L. Sci. Rep. 10:4916. doi: 10.1038/s41598-020-61846-x, 32188930 PMC7080738

[ref160] Nag M. Lahiri D. Ghosh A. Das D. Ray R. R. (2021). “Quorum sensing,” in Biofilm-Mediated Diseases: Causes and Controls, eds. Ray R. R. Nag M. Lahiri D. (Singapore: Springer), 27–49.

[ref161] Nagy R. Drissner D. Amrhein N. Jakobsen I. Bucher M. (2009). Mycorrhizal phosphate uptake pathway in tomato is phosphorus-repressible and transcriptionally regulated. New Phytol. 181, 950–959. doi: 10.1111/j.1469-8137.2008.02721.x, 19140941

[ref162] Nagy R. Karandashov V. Chague V. Kalinkevich K. Tamasloukht M. Xu G. . (2005). The characterization of novel mycorrhiza-specific phosphate transporters from *Lycopersicon esculentum* and *Solanum tuberosum* uncovers functional redundancy in symbiotic phosphate transport in solanaceous species. Plant J. 42, 236–250. doi: 10.1111/j.1365-313X.2005.02364.x, 15807785

[ref163] Naik B. Kumar V. Rizwanuddin S. Chauhan M. Choudhary M. Gupta A. K. . (2023). Genomics, proteomics, and metabolomics approaches to improve abiotic stress tolerance in tomato plant. Int. J. Mol. Sci. 24:3025. doi: 10.3390/ijms24033025, 36769343 PMC9918255

[ref164] Nanjundappa A. Bagyaraj D. J. Saxena A. K. Kumar M. Chakdar H. (2019). Interaction between arbuscular mycorrhizal fungi and *Bacillus* spp. in soil enhancing growth of crop plants. Fungal Biol. Biotechnol. 6:23. doi: 10.1186/s40694-019-0086-5, 31798924 PMC6882151

[ref165] Niu B. Wang W. Yuan Z. Sederoff R. R. Sederoff H. Chiang V. L. . (2020). Microbial interactions within multiple-strain biological control agents impact soil-borne plant disease. Front. Microbiol. 11:585404. doi: 10.3389/fmicb.2020.585404, 33162962 PMC7581727

[ref166] Nivetha N. Lavanya A. Vikram K. Asha A. Sruthi K. Bandeppa S. . (2021). “PGPR-mediated regulation of antioxidants: prospects for abiotic stress management in plants,” in Antioxidants in Plant-Microbe Interaction, eds. Singh H. B. Vaishnav A. Sayyed R. (Singapore: Springer), 471–498.

[ref167] Nonaka S. Ezura H. (2014). Plant–*Agrobacterium* interaction mediated by ethylene and super-*Agrobacterium* conferring efficient gene transfer. Front. Plant Sci. 5:681. doi: 10.3389/fpls.2014.00681, 25520733 PMC4253739

[ref168] Numan M. Bashir S. Khan Y. Mumtaz R. Shinwari Z. K. Khan A. L. . (2018). Plant growth promoting bacteria as an alternative strategy for salt tolerance in plants: a review. Microbiol. Res. 209, 21–32. doi: 10.1016/j.micres.2018.02.003, 29580619

[ref169] Nwachukwu B. C. Ayangbenro A. S. Babalola O. O. (2021). Elucidating the rhizosphere associated bacteria for environmental sustainability. Agriculture 11:75. doi: 10.3390/agriculture11010075

[ref170] Olaniyan F. T. Alori E. T. Adekiya A. O. Ayorinde B. B. Daramola F. Y. Osemwegie O. O. . (2022). The use of soil microbial potassium solubilizers in potassium nutrient availability in soil and its dynamics. Ann. Microbiol. 72:45. doi: 10.1186/s13213-022-01701-8

[ref171] Ordookhani K. Khavazi K. Moezzi A. Rejali F. (2010). Influence of PGPR and AMF on antioxidant activity, lycopene and potassium contents in tomato. Afr. J. Agric. Res. 5, 1108–1116.

[ref172] Osakabe Y. Osakabe K. Shinozaki K. Tran L. S. (2014). Response of plants to water stress. Front. Plant Sci. 5:86. doi: 10.3389/fpls.2014.00086, 24659993 PMC3952189

[ref173] Ouziad F. Wilde P. Schmelzer E. Hildebrandt U. Bothe H. (2006). Analysis of expression of aquaporins and Na^+^/H^+^ transporters in tomato colonized by arbuscular mycorrhizal fungi and affected by salt stress. Environ. Exp. Bot. 57, 177–186. doi: 10.1016/j.envexpbot.2005.05.011

[ref174] Panpatte D. G. Jhala Y. K. Vyas R. V. (2020). “Signaling pathway of induced systemic resistance,” in Molecular Aspects of Plant Beneficial Microbes in Agriculture, ed. Sarma M. K. (Amsterdam: Elsevier), 133–141.

[ref175] Pantoja-Guerra M. Valero-Valero N. Ramírez C. A. (2023). Total auxin level in the soil–plant system as a modulating factor for the effectiveness of PGPR inocula: a review. Chem. Biol. Technol. Agric. 10:6. doi: 10.1186/s40538-022-00370-8

[ref176] Pattnaik D. Dash D. Mishra A. Padhiary A. K. Dey P. Dash G. K. (2021). “Emerging roles of osmoprotectants in alleviating abiotic stress response under changing climatic conditions,” in Climate Impacts on Sustainable Natural Resource Management, eds. Kumar P. Meena R. S. Jat M. L. (Hoboken, NJ: Wiley), 303–324.

[ref177] Peleg Z. Blumwald E. (2011). Hormone balance and abiotic stress tolerance in crop plants. Curr. Opin. Plant Biol. 14, 290–295. doi: 10.1016/j.pbi.2011.02.001, 21377404

[ref178] Pérez-de-Luque A. Tille S. Johnson I. Pascual-Pardo D. Ton J. Cameron D. D. (2017). The interactive effects of arbuscular mycorrhiza and plant growth-promoting rhizobacteria synergistically enhance host plant defences against pathogens. Sci. Rep. 7:16409. doi: 10.1038/s41598-017-16697-4, 29180695 PMC5703727

[ref179] Pieterse C. M. Leon-Reyes A. Van der Ent S. Van Wees S. C. (2009). Networking by small-molecule hormones in plant immunity. Nat. Chem. Biol. 5, 308–316. doi: 10.1038/nchembio.164, 19377457

[ref180] Pieterse C. M. Zamioudis C. Berendsen R. L. Weller D. M. Van Wees S. C. Bakker P. A. (2014). Induced systemic resistance by beneficial microbes. Annu. Rev. Phytopathol. 52, 347–375. doi: 10.1146/annurev-phyto-082712-102340, 24906124

[ref181] Pozo M. J. Azcón-Aguilar C. (2007). Unraveling mycorrhiza-induced resistance. Curr. Opin. Plant Biol. 10, 393–398. doi: 10.1016/j.pbi.2007.05.004, 17658291

[ref182] Primieri S. Magnoli S. M. Koffel T. Stürmer S. L. Bever J. D. (2022). Perennial, but not annual legumes synergistically benefit from infection with arbuscular mycorrhizal fungi and rhizobia: a meta-analysis. New Phytol. 233, 505–514. doi: 10.1111/nph.17787, 34626495 PMC9298428

[ref183] Qin J. Qin Z. Ni G. Xie M. Zhou D. Wang G. . (2024). Advances in the separate functions or cross-kingdom interactions of AMF and PGPR in enhancing plant salt tolerance. J. Plant Nutr. Fertil. 30, 1354–1366. doi: 10.11674/zwyf.2024232

[ref184] Raaijmakers J. M. Mazzola M. (2012). Diversity and natural functions of antibiotics produced by beneficial and plant pathogenic bacteria. Annu. Rev. Phytopathol. 50, 403–424. doi: 10.1146/annurev-phyto-081211-172908, 22681451

[ref185] Rai G. K. Kumar P. Choudhary S. M. Kosser R. Khanday D. M. Choudhary S. . (2022). Biomimetic strategies for developing abiotic stress-tolerant tomato cultivars: an overview. Plants 12:86. doi: 10.3390/plants12010086, 36616215 PMC9823378

[ref186] Raja Gopalan N. S. Nikhil P. T. Sharma R. Mohapatra S. (2023). The use of microbes as a combative strategy for alleviation of abiotic and biotic stresses. Unravelling Plant-Microbe Synergy, Academic Press. 175–193.

[ref187] Rasheed Y. Khalid F. Ashraf H. Asif K. Maqsood M. F. Naz N. . (2024). Enhancing plant stress resilience with osmolytes and nanoparticles. J. Soil Sci. Plant Nutr. 24, 1871–1906. doi: 10.1007/s42729-024-01821-x

[ref188] Redecker D. Schüssler A. Stockinger H. Stürmer S. L. Morton J. B. Walker C. (2013). An evidence-based consensus for the classification of arbuscular mycorrhizal fungi (Glomeromycota). Mycorrhiza 23, 515–531. doi: 10.1007/s00572-013-0486-y, 23558516

[ref189] Reginato M. Cenzano A. M. Arslan I. Furlán A. Varela C. Cavallin V. . (2021). Na2SO4 and NaCl salts differentially modulate the antioxidant systems in the highly stress tolerant halophyte *Prosopis strombulifera*. Plant Physiol. Biochem. 167, 748–762. doi: 10.1016/j.plaphy.2021.09.003 34509937

[ref190] Rivero J. Álvarez D. Flors V. Azcón-Aguilar C. Pozo M. J. (2018). Root metabolic plasticity underlies functional diversity in mycorrhiza-enhanced stress tolerance in tomato. New Phytol. 220, 1322–1336. doi: 10.1111/nph.15295, 29982997

[ref191] Rivero J. Gamir J. Aroca R. Pozo M. J. Flors V. (2015). Metabolic transition in mycorrhizal tomato roots. Front. Microbiol. 6:598. doi: 10.3389/fmicb.2015.00598, 26157423 PMC4477175

[ref192] Rodríguez P. Dell'Amico J. Morales D. Blanco M. J. S. Alarcón J. J. (1997). Effects of salinity on growth, shoot water relations and root hydraulic conductivity in tomato plants. J. Agric. Sci. 128, 439–444.

[ref193] Roșca M. Mihalache G. Stoleru V. (2023). Tomato responses to salinity stress: From morphological traits to genetic changes. Front. Plant Sci. 14:1118383. doi: 10.3389/fpls.2023.1118383 36909434 PMC10000760

[ref194] Roychoudhry S. Kepinski S. (2022). Auxin in root development. Cold Spring Harb. Perspect. Biol. 14:a039933. doi: 10.1101/cshperspect.a039933, 34312248 PMC9121899

[ref195] Ruiz-Lozano J. M. Aroca R. Zamarreño Á. M. Molina S. Andreo-Jiménez B. Porcel R. . (2016). Arbuscular mycorrhizal symbiosis induces strigolactone biosynthesis under drought and improves drought tolerance in lettuce and tomato. Plant Cell Environ. 39, 441–452. doi: 10.1111/pce.12631, 26305264

[ref196] Sagar A. Rathore P. Ramteke P. W. Ramakrishna W. Reddy M. S. Pecoraro L. (2021). Plant growth promoting rhizobacteria, arbuscular mycorrhizal fungi and their synergistic interactions to counteract the negative effects of saline soil on agriculture: key macromolecules and mechanisms. Microorganisms 9:1491. doi: 10.3390/microorganisms9071491, 34361927 PMC8307984

[ref197] Santander C. Sanhueza M. Olave J. Borie F. Valentine A. Cornejo P. (2019). Arbuscular mycorrhizal colonization promotes the tolerance to salt stress in lettuce plants through an efficient modification of ionic balance. J. Soil Sci. Plant Nutr. 19, 321–331. doi: 10.1007/s42729-019-00032-z

[ref198] Santos-Medellín C. Edwards J. Liechty Z. Nguyen B. Sundaresan V. (2017). Drought stress results in a compartment-specific restructuring of the rice root-associated microbiomes. MBio 8:e00764-17. doi: 10.1128/mBio.00764-17, 28720730 PMC5516253

[ref199] Sarwar M. J. Zahir Z. A. Asghar H. N. Khaliq A. (2022). Interaction of cadmium tolerant plant growth promoting rhizobacteria and organic amendments to suppress cadmium uptake in tomato. Pak. J. Agric. Sci. 59, 737–747. doi: 10.21162/PAKJAS/22.82

[ref200] Savastano N. Bais H. (2024). Synergism or antagonism: do arbuscular mycorrhizal fungi and plant growth-promoting rhizobacteria work together to benefit plants? Int. J. Plant Biol. 15, 944–958. doi: 10.3390/ijpb15040067

[ref201] Schouteden N. De Waele D. Panis B. Vos C. M. (2015). Arbuscular mycorrhizal fungi for the biocontrol of plant-parasitic nematodes: a review of the mechanisms involved. Front. Microbiol. 6:1280. doi: 10.3389/fmicb.2015.01280, 26635750 PMC4646980

[ref202] Shawky A. Hatawsh A. Al-Saadi N. Farzan R. Eltawy N. Francis M. . (2024). Revolutionizing tomato cultivation: CRISPR/Cas9 mediated biotic stress resistance. Plants 13:2269. doi: 10.3390/plants13162269, 39204705 PMC11360581

[ref203] Shi Y. Zhang Z. Yan Z. Chu H. Luo C. (2025). Tomato mitogen-activated protein kinase: mechanisms of adaptation in response to biotic and abiotic stresses. Front. Plant Sci. 16:1533248. doi: 10.3389/fpls.2025.1533248, 39963529 PMC11830615

[ref204] Singh R. K. Singh P. Li H. B. Song Q. Q. Guo D. J. Solanki M. K. . (2020). Diversity of nitrogen-fixing rhizobacteria associated with sugarcane: a comprehensive study of plant-microbe interactions for growth enhancement in Saccharum spp. BMC Plant Biol. 20:220. doi: 10.1186/s12870-020-02400-9, 32423383 PMC7236179

[ref205] Smith S. E. Smith F. A. (2011). Roles of arbuscular mycorrhizas in plant nutrition and growth: new paradigms from cellular to ecosystem scales. Annu. Rev. Plant Biol. 62, 227–250. doi: 10.1146/annurev-arplant-042110-103846, 21391813

[ref206] Soliveres S. Maestre F. T. Bowker M. A. Torices R. Quero J. L. García-Gómez M. . (2014). Functional traits determine plant co-occurrence more than environment or evolutionary relatedness in global drylands. *Perspect*. *Plant Ecol. Evol. Syst.* 16, 164–173. doi: 10.1016/j.ppees.2014.05.001, 25914604 PMC4407970

[ref207] Soltabayeva A. Dauletova N. Serik S. Sandybek M. Omondi J. O. Kurmanbayeva A. . (2022). Receptor-like kinases (LRR-RLKs) in response of plants to biotic and abiotic stresses. Plants 11:2660. doi: 10.3390/plants11192660, 36235526 PMC9572924

[ref208] Song Y. Chen D. Lu K. Sun Z. Zeng R. (2015). Enhanced tomato disease resistance primed by arbuscular mycorrhizal fungus. Front. Plant Sci. 6:786. doi: 10.3389/fpls.2015.00786, 26442091 PMC4585261

[ref209] Song Y. Y. Zeng R. S. Xu J. F. Li J. Shen X. Yihdego W. G. (2010). Interplant communication of tomato plants through underground common mycorrhizal networks. PLoS One 5:e13324. doi: 10.1371/journal.pone.0013324, 20967206 PMC2954164

[ref210] Sood M. Kapoor D. Kumar V. Kalia N. Bhardwaj R. Sidhu G. P. S. . (2021). Mechanisms of plant defense under pathogen stress: A review. Curr. Protein Pept. Sci. 22, 376–395. doi: 10.2174/1389203722666210125122827, 33550968

[ref211] Sosnowski J. Truba M. Vasileva V. (2023). The impact of auxin and cytokinin on the growth and development of selected crops. Agriculture 13:724. doi: 10.3390/agriculture13030724

[ref212] Srinivasarao C. Shanker A. K. Kundu S. Reddy S. (2016). Chlorophyll fluorescence induction kinetics and yield responses in rainfed crops with variable potassium nutrition in K deficient semi-arid alfisols. J. Photochem. Photobiol. B Biol. 160, 86–95. doi: 10.1016/j.jphotobiol.2016.03.052, 27101276

[ref213] Srivastava S. Ranjan M. Bano N. Asif M. H. Srivastava S. (2023). Comparative transcriptome analysis reveals the phosphate starvation alleviation mechanism of phosphate accumulating *Pseudomonas putida* in *Arabidopsis thaliana*. Sci. Rep. 13:4918. doi: 10.1038/s41598-023-31154-1, 36966146 PMC10039930

[ref214] Stringlis I. A. Yu K. Feussner K. de Jonge R. Van Bentum S. Van Verk M. C. . (2018). MYB72-dependent coumarin exudation shapes root microbiome assembly to promote plant health. Proc. Natl. Acad. Sci. USA 115, E5213–E5222. doi: 10.1073/pnas.1722335115, 29686086 PMC5984513

[ref216] Subramanian K. S. Santhanakrishnan P. Balasubramanian P. (2006). Responses of field grown tomato plants to arbuscular mycorrhizal fungal colonization under varying intensities of drought stress. Sci. Hortic. 107, 245–253. doi: 10.1016/j.scienta.2005.07.006

[ref215] Su L. Zhang L. Nie D. Kuramae E. E. Shen B. Shen Q. (2020). Bacterial tomato pathogen *Ralstonia solanacearum* invasion modulates rhizosphere compounds and facilitates the cascade effect of fungal pathogen Fusarium solani. Microorganisms 8:806. doi: 10.3390/microorganisms8060806, 32471167 PMC7356623

[ref217] Sun D. Shang X. Cao H. Lee S.-J. Wang L. Gan Y. . (2024). Physio-biochemical mechanisms of arbuscular mycorrhizal fungi enhancing plant resistance to abiotic stress. Agriculture 14:2361. doi: 10.3390/agriculture14122361

[ref218] Sun Y. Wang C. Chen H. Y. H. Ruan H. (2020). Response of plants to water stress: A meta-analysis. Front. Plant Sci. 11:978. doi: 10.3389/fpls.2020.00978, 32676096 PMC7333662

[ref219] Tahiri A. Meddich A. Raklami A. Alahmad A. Bechtaoui N. Anli M. . (2022). Assessing the potential role of compost, PGPR, and AMF in improving tomato plant growth, yield, fruit quality, and water stress tolerance. J. Soil Sci. Plant Nutr. 22, –764. doi: 10.1007/s42729-021-00684-w

[ref220] Tanveer K. Gilani S. Hussain Z. Ishaq R. Adeel M. Ilyas N. (2020). Effect of salt stress on tomato plant and the role of calcium. J. Plant Nutr. 43, 28–35. doi: 10.1080/01904167.2019.1659324

[ref221] Timm C. M. Carter K. R. Carrell A. A. Jun S. R. Jawdy S. S. Vélez J. M. . (2018). Abiotic stresses shift belowground Populus-associated bacteria toward a core stress microbiome. mSystems 3:e00070-17. doi: 10.1128/mSystems.00070-17, 29404422 PMC5781258

[ref222] Timofeeva A. M. Galyamova M. R. Sedykh S. E. (2022). Bacterial siderophores: classification, biosynthesis, perspectives of use in agriculture. Plants 11:3065. doi: 10.3390/plants11223065, 36432794 PMC9694258

[ref223] Toju H. Peay K. G. Yamamichi M. Narisawa K. Hiruma K. Naito K. . (2018). Core microbiomes for sustainable agroecosystems. Nat. Plants 4, 247–257. doi: 10.1038/s41477-018-0139-4, 29725101

[ref224] Ton J. Flors V. Mauch-Mani B. (2009). The multifaceted role of ABA in disease resistance. Trends Plant Sci. 14, 310–317. doi: 10.1016/j.tplants.2009.03.006, 19443266

[ref225] Toro M. Azcon R. Barea J. (1997). Improvement of arbuscular mycorrhizal development by inoculation of soil with phosphate-solubilizing rhizobacteria to improve rock phosphate bioavailability (32P) and nutrient cycling. Appl. Environ. Microbiol. 63, 4408–4412. doi: 10.1128/aem.63.11.4408-4412.1997, 16535730 PMC1389286

[ref226] Tripathi P. Subedi S. Khan A. L. Chung Y.-S. Kim Y. (2021). Silicon effects on the root system of diverse crop species using root phenotyping technology. Plants 10:885. doi: 10.3390/plants10050885, 33924781 PMC8145683

[ref227] Trivedi P. Leach J. E. Tringe S. G. Sa T. Singh B. K. (2020). Plant–microbiome interactions: from community assembly to plant health. Nat. Rev. Microbiol. 18, 607–621. doi: 10.1038/s41579-020-0412-1, 32788714

[ref228] Trouvelot S. Bonneau L. Redecker D. van Tuinen D. Adrian M. Wipf D. (2015). Arbuscular mycorrhiza symbiosis in viticulture: a review. Agron. Sustain. Dev. 35, 1449–1467. doi: 10.1007/s13593-015-0329-7

[ref229] Tufail T. Bader Ul Ain H. Noreen S. Ikram A. Arshad M. T. Abdullahi M. A. (2024). Nutritional benefits of lycopene and beta-carotene: a comprehensive overview. Food Sci. Nutr. 12, 8715–8741. doi: 10.1002/fsn3.4502, 39619948 PMC11606860

[ref230] Umar W. Ayub M. A. Rehman M. Z. U. Ahmad H. R. Farooqi Z. U. R. Shahzad A. . (2020). “Nitrogen and phosphorus use efficiency in agroecosystems,” in Resources Use Efficiency in Agriculture, eds. Kumar S. Meena R. S. Jhariya M. K. (Singapore: Springer), 213–257.

[ref231] Umer M. Anwar N. Mubeen M. Li Y. Ali A. Alshaharni M. O. . (2025). Roles of arbuscular mycorrhizal fungi in plant growth and disease management for sustainable agriculture. Front. Microbiol. 16:1616273. doi: 10.3389/fmicb.2025.1616273, 40778206 PMC12329470

[ref235] Vandana U. K. Rajkumari J. Singha L. P. Satish L. Alavilli H. Sudheer P. D. V. N. . (2021). The endophytic microbiome as a hotspot of synergistic interactions, with prospects of plant growth promotion. Biology 10:101. doi: 10.3390/biology10020101, 33535706 PMC7912845

[ref232] Van der Ent S. Van Wees S. C. Pieterse C. M. (2009). Jasmonate signaling in plant interactions with resistance-inducing beneficial microbes. Phytochemistry 70, 1581–1588. doi: 10.1016/j.phytochem.2009.06.009, 19712950

[ref233] van der Heijden M. G. Streitwolf-Engel R. Riedl R. Siegrist S. Neudecker A. Ineichen K. . (2006). The mycorrhizal contribution to plant productivity, plant nutrition and soil structure in experimental grassland. New Phytol. 172, 739–752. doi: 10.1111/j.1469-8137.2006.01862.x, 17096799

[ref234] van Zelm E. Zhang Y. Testerink C. (2020). Salt tolerance mechanisms of plants. Annu. Rev. Plant Biol. 71, 403–433. doi: 10.1146/annurev-arplant-050718-100005, 32167791

[ref236] Verma P. K. Verma S. Pandey N. (2022). Root system architecture in rice: impacts of genes, phytohormones and root microbiota. Biotech 12:239. doi: 10.1007/s13205-022-03299-9, 36016841 PMC9395555

[ref237] Vivas A. Marulanda A. Ruiz-Lozano J. M. Barea J. M. Azcón R. (2003). Influence of a Bacillus sp. on physiological activities of two arbuscular mycorrhizal fungi and on plant responses to PEG-induced drought stress. Mycorrhiza 13, 249–256. doi: 10.1007/s00572-003-0223-z, 14593518

[ref238] Vocciante M. Grifoni M. Fusini D. Petruzzelli G. Franchi E. (2022). The role of plant growth-promoting rhizobacteria (PGPR) in mitigating plant's environmental stresses. Appl. Sci. 12:1231. doi: 10.3390/app12031231

[ref239] Volpe V. Chitarra W. Cascone P. Volpe M. G. Bartolini P. Moneti G. . (2018). The association with two different arbuscular mycorrhizal fungi differently affects water stress tolerance in tomato. Front. Plant Sci. 9:1480. doi: 10.3389/fpls.2018.01480, 30356724 PMC6189365

[ref240] Volpin H. Elkind Y. Okon Y. Kapulnik Y. (1994). A vesicular arbuscular mycorrhizal fungus (Glomus intraradix) induces a defense response in alfalfa roots. Plant Physiol. 104, 683–689. doi: 10.1104/pp.104.2.683, 12232119 PMC159247

[ref241] Vos C. M. Tesfahun A. N. Panis B. De Waele D. Elsen A. (2012). Arbuscular mycorrhizal fungi induce systemic resistance in tomato against the sedentary nematode *Meloidogyne incognita* and the migratory nematode *Pratylenchus penetrans*. Appl. Soil Ecol. 61, 1–6. doi: 10.1016/j.apsoil.2012.04.007

[ref242] Wahab A. Batool F. Abdi G. Muhammad M. Ullah S. Zaman W. (2025). Role of plant growth-promoting rhizobacteria in sustainable agriculture: Addressing environmental and biological challenges. J. Plant Physiol. 307:154455. doi: 10.1016/j.jplph.2025.154455, 40037066

[ref243] Wahab A. Batool F. Muhammad M. Zaman W. Mikhlef R. M. Naeem M. (2023a). Current knowledge, research progress, and future prospects of phyto-synthesized nanoparticles interactions with food crops under induced drought stress. Sustainability 15:14792. doi: 10.3390/su152014792

[ref244] Wahab A. Muhammad M. Munir A. Abdi G. Zaman W. Ayaz A. . (2023b). Role of arbuscular mycorrhizal fungi in regulating growth, enhancing productivity, and potentially influencing ecosystems under abiotic and biotic stresses. Plants 12:3102. doi: 10.3390/plants12173102, 37687353 PMC10489935

[ref246] Wang G. Jin Z. George T. S. Feng G. Zhang L. (2023). Arbuscular mycorrhizal fungi enhance plant phosphorus uptake through stimulating hyphosphere soil microbiome functional profiles for phosphorus turnover. New Phytol. 238, 2578–2593. doi: 10.1111/nph.18772, 36694293

[ref245] Wang H. Hao Z. Zhang X. Xie W. Chen B. (2022). Arbuscular mycorrhizal fungi induced plant resistance against Fusarium wilt in jasmonate biosynthesis defective mutant and wild type of tomato. J. Fungi 8:422. doi: 10.3390/jof8050422, 35628678 PMC9146357

[ref9003] Wei G. Kloepper J. W. Tuzun S. Induction of systemic resistance of cucumber to Colletotrichum orbiculare by select strains of plant growth promoting rhizobacteria. Phytopathology. (1991), 81, 15081512. doi: 10.1094/Phyto-81-1508

[ref247] Wei Z. Yang T. Friman V. P. Xu Y. Shen Q. Jousset A. (2015). Trophic network architecture of root-associated bacterial communities determines pathogen invasion and plant health. Nat. Commun. 6:8413. doi: 10.1038/ncomms9413, 26400552 PMC4598729

[ref248] Wilkes T. I. Warner D. J. Edmonds-Brown V. Davies K. G. (2020). Species-specific interactions of Bacillus innocula and arbuscular mycorrhizal fungi symbiosis with winter wheat. Microorganisms 8:1795. doi: 10.3390/microorganisms8111795, 33207834 PMC7697830

[ref249] Wipf D. Krajinski F. van Tuinen D. Recorbet G. Courty P. E. (2019). Trading on the arbuscular mycorrhiza market: from arbuscules to common mycorrhizal networks. New Phytol. 223, 1127–1142. doi: 10.1111/nph.15775, 30843207

[ref250] Xiong C. Lu Y. (2022). Microbiomes in agroecosystem: Diversity, function and assembly mechanisms. Environ. Microbiol. Rep. 14, 833–849. doi: 10.1111/1758-2229.13126, 36184075

[ref9002] Xu J. Xing X. J. Tian Y. S. Peng R. H. Xue Y. Zhao W. (2015). Transgenic arabidopsis plants expressing tomato glutathione S-transferase showed enhanced resistance to salt and drought stress. PloS one, 10, e0136960. doi:10.1371/journal.pone.0136960 26327625 PMC4556630

[ref251] Xu L. Li T. Wu Z. Feng H. Yu M. Zhang X. . (2018). Arbuscular mycorrhiza enhances drought tolerance of tomato plants by regulating the 14-3-3 genes in the ABA signaling pathway. Appl. Soil Ecol. 125, 213–221. doi: 10.1016/j.apsoil.2018.01.012

[ref253] Yurtseven E. Kesmez G. D. Unlukara A. (2005). The effects of water salinity and potassium levels on yield, fruit quality and water consumption of a native central Anatolian tomato species (*Lycopersicon esculentum*). Agric. Water Manag. 78, 128–135. doi: 10.1016/j.agwat.2005.04.018

[ref252] Yu Y. Gui Y. Li Z. Jiang C. Guo J. Niu D. (2022). Induced systemic resistance for improving plant immunity by beneficial microbes. Plants 11:386. doi: 10.3390/plants11030386, 35161366 PMC8839143

[ref254] Zamioudis C. Pieterse C. M. (2012). Modulation of host immunity by beneficial microbes. Mol. Plant-Microbe Interact. 25, 139–150. doi: 10.1094/MPMI-06-11-0179, 21995763

[ref255] Zandi P. Schnug E. (2022). Reactive oxygen species, antioxidant responses and implications from a microbial modulation perspective. Biology 11:155. doi: 10.3390/biology11020155, 35205022 PMC8869449

[ref256] Zare M. Ordookhani K. Alizadeh O. (2011). Effects of PGPR and AMF on growth of two bred cultivars of tomato. Adv. Environ. Biol. 5, 2177–2181.

[ref257] Zelezniak A. Andrejev S. Ponomarova O. Mende D. R. Bork P. Patil K. R. (2015). Metabolic dependencies drive species co-occurrence in diverse microbial communities. Proc. Natl. Acad. Sci. USA 112, 6449–6454. doi: 10.1073/pnas.1421834112, 25941371 PMC4443341

[ref258] Zeng Q. Hu H. W. Ge A. H. Xiong C. Zhai C. C. Duan G. L. . (2025). Plant–microbiome interactions and their impacts on plant adaptation to climate change. J. Integr. Plant Biol. 67, 826–844. doi: 10.1111/jipb.13863, 39981843

[ref260] Zhang J. Zhou J. M. (2010). Plant immunity triggered by microbial molecular signatures. Mol. Plant 3, 783–793. doi: 10.1093/mp/ssq035, 20713980

[ref259] Zhang J. Y. Cruz De Carvalho M. H. Torres-Jerez I. Kang Y. Allen S. N. Huhman D. V. . (2014). Global reprogramming of transcription and metabolism in *Medicago truncatula* during progressive drought and after rewatering. Plant Cell Environ. 37, 2553–2576. doi: 10.1111/pce.12328 24661137 PMC4260174

[ref261] Zhang L. Zuluaga M. Y. A. Pii Y. Barone A. Amaducci S. Miras-Moreno B. . (2023). A Pseudomonas plant growth promoting rhizobacterium and arbuscular mycorrhiza differentially modulate the growth, photosynthetic performance, nutrients allocation, and stress response mechanisms triggered by a mild zinc and cadmium stress in tomato. Plant Sci. 337:111873. doi: 10.1016/j.plantsci.2023.111873, 37739018

[ref262] Zhao S. Li M. Ren X. Wang C. Sun X. Sun M. . (2024). Enhancement of broad-spectrum disease resistance in wheat through key genes involved in systemic acquired resistance. Front. Plant Sci. 15:1355178. doi: 10.3389/fpls.2024.1355178, 38463563 PMC10921362

[ref265] Zhou L. Zhou L. Wu H. Li J. Kong L. Yang H. (2024). Effects of applying biochar on soil cadmium immobilisation and cadmium pollution control in lettuce (*Lactuca sativa* L.). Agriculture 14:1068. doi: 10.3390/agriculture14071068

[ref264] Zhou W. Zhang M. M. Tao K. Z. Zhu X. C. (2022). Effects of arbuscular mycorrhizal fungi and plant growth-promoting rhizobacteria on growth and reactive oxygen metabolism of tomato fruits under low saline conditions. Biocell 46, 2575–2582. doi: 10.32604/biocell.2022.021910

[ref263] Zhou X. Zhang X. Ma C. Wu F. Jin X. Dini-Andreote F. . (2022). Biochar amendment reduces cadmium uptake by stimulating cadmium-resistant PGPR in tomato rhizosphere. Chemosphere 307:136138. doi: 10.1016/j.chemosphere.2022.136138, 36002065

[ref266] Zhu L. Huang J. Lu X. Zhou C. (2022). Development of plant systemic resistance by beneficial rhizobacteria: Recognition, initiation, elicitation and regulation. Front. Plant Sci. 13:952397. doi: 10.3389/fpls.2022.952397, 36017257 PMC9396261

[ref267] Zia R. Nawaz M. S. Siddique M. J. Hakim S. Imran A. (2021). Plant survival under drought stress: Implications, adaptive responses, and integrated rhizosphere management strategy for stress mitigation. Microbiol. Res. 242:126626. doi: 10.1016/j.micres.2020.126626, 33189069

